# Central carbon flux controls growth/damage balance for *Streptococcus pyogenes*

**DOI:** 10.1371/journal.ppat.1011481

**Published:** 2023-06-29

**Authors:** Joseph A. Merriman, Wei Xu, Michael G. Caparon

**Affiliations:** Department of Molecular Microbiology, Washington University School of Medicine, St. Louis, Missouri, United States of America; Lunds universitet Medicinska fakulteten, SWEDEN

## Abstract

Microbial pathogens balance growth against tissue damage to achieve maximum fitness. Central carbon metabolism is connected to growth, but how it influences growth/damage balance is largely unknown. Here we examined how carbon flux through the exclusively fermentative metabolism of the pathogenic lactic acid bacterium *Streptococcus pyogenes* impacts patterns of growth and tissue damage. Using a murine model of soft tissue infection, we systematically examined single and pair-wise mutants that constrained carbon flux through the three major pathways that *S*. *pyogenes* employs for reduction of the glycolytic intermediate pyruvate, revealing distinct disease outcomes. Its canonical lactic acid pathway (via lactate dehydrogenase) made a minimal contribution to virulence. In contrast, its two parallel pathways for mixed-acid fermentation played important, but non-overlapping roles. Anaerobic mixed acid fermentation (via pyruvate formate lyase) was required for growth in tissue, while aerobic mixed-acid pathway (via pyruvate dehydrogenase) was not required for growth, but instead regulated levels of tissue damage. Infection of macrophages *in vitro* revealed that pyruvate dehydrogenase was required to prevent phagolysosomal acidification, which altered expression of the immunosuppressive cytokine IL-10. Infection of IL-10 deficient mice confirmed that the ability of aerobic metabolism to regulate levels of IL-10 plays a key role in the ability of *S*. *pyogenes* to modulate levels of tissue damage. Taken together, these results show critical non-overlapping roles for anaerobic and aerobic metabolism in soft tissue infection and provide a mechanism for how oxygen and carbon flux act coordinately to regulate growth/damage balance. Therapies targeting carbon flux could be developed to mitigate tissue damage during severe *S*. *pyogenes* infection.

## Author summary

The Gram-positive bacterium *Streptococcus pyogenes* causes a wide range of diseases that differ significantly in tissue damage, including necrotizing fasciitis (flesh-eating disease) which can cause severe destruction of skin and underlying tissue. Treatment of severe disease often requires surgical intervention, including amputation, to remove infected tissue. How *S*. *pyogenes* can cause either mild or tissue-destructive infections is not well-understood. In this study, we examined how different streptococcal energy-producing pathways that convert host-derived carbon sources into cellular energy influence the levels of tissue damage that *S*. *pyogenes* can produce. Using a mouse model of cutaneous infection, we found that two parallel pathways for mixed-acid fermentation play important and non-overlapping roles. One pathway that is inhibited by oxygen (using the enzyme pyruvate formate lyase) is required for *S*. *pyogenes* to grow in tissue. The second requires oxygen (using the enzyme pyruvate dehydrogenase), is not required for growth, but instead regulates levels of tissue damage by supporting the ability of *S*. *pyogenes* to manipulate host cytokine production to control the immune response. Understanding how metabolism influences tissue damage helps to explain how *S*. *pyogenes* diseases can differ in severity and identifies new targets for treatments to limit tissue damage in severe infections.

## Introduction

The diverse collection of Gram-positive bacterial genera known as the lactic acid bacteria are prominent members of both the commensal and pathogenic flora [1,2]. Their eponymous characteristic derives from an exclusively fermentative metabolism that under ideal growth conditions uses Embden-Meyerhof-Parnas glycolysis to generate pyruvate, which is subsequently converted to lactate as its principal end-product [3–6]. Less-well appreciated is the metabolic plasticity of these bacteria, most of which possess alternative pathways for pyruvate reduction leading to the generation of additional end-products, including CO_2_, ethanol, formate, acetate and several other metabolites [4–6]. Work in the microbiome space indicates that many of these metabolites help shape health and disease through immunomodulatory effects [7,8]. However, for the pathogenic lactic acid bacteria, such as *Streptococcus pyogenes* (group A streptococcus), how these alternative pathways support the development of disease is generally not well-understood.

A pathogen of global significance, *S*. *pyogenes* is responsible for approximately 500,000 deaths per year despite its continued susceptibility to beta-lactam antibiotics [9,10]. Diversity of infection sites drives this global burden, to cause diseases ranging from mild and self-limiting (impetigo, pharyngitis), to severe and life-threatening (cellulitis, necrotizing fasciitis) and include serious post-infection sequelae (rheumatic fever, acute glomerulonephritis) [10]. It is likely that the heterogenous host niches infected by *S*. *pyogenes* differ significantly in the availability of substrates supportive of *S*. *pyogenes* growth. Considering that the substrates available in any single niche will change over the course of infection, metabolic plasticity, including alternative pathways for pyruvate reduction, likely play a critical role in promoting fitness in these temporally and metabolically dynamic environments.

It is well-established that metabolic substrate availability directly influences expression of *S*. *pyogenes* virulence factors [11,12]. For example, comparisons of the *S*. *pyogenes* transcriptome between organisms recovered from various animal models of infection has revealed both temporal and tissue-specific patterns of gene expression [13–15]. When examined *in vitro*, many of these patterns can be reproduced by alterations to the composition of media, including changes in pH and the concentration of glucose vs alternative carbon substrates [16]. These two conditions are likely related, as *S*. *pyogenes’* choice of carbon substrate will influence the composition of the organic acid end-products its metabolism will produce [17], resulting in a significant remodeling of its local tissue environment though changing pH, oxygen tension and the accumulation of immunomodulatory metabolites. Since different tissue compartments vary in carbon source profile, both the preferred carbon source, as well as the hierarchical use of alternative carbon sources are likely major host-derived cues that influence the expression of the virulence transcriptome, altering both its configuration and its temporal dynamics.

This model is supported by analysis of several global regulators of virulence gene transcription whose function is intimately intertwined with metabolism. These include Mga, LacD.1, RopB, CodY and CcpA, whose major signaling inputs include the chemical characteristics of specific carbon sources, temporal changes in external pH and carbon flow through central metabolic pathways [12]. Mutations in many of these regulators can have a profound influence on the pathogen’s growth/damage balance, a process by which the pathogen balances its growth rate against the degree to which host tissues are damaged in order to achieve optimal fitness [18]. The DNA-binding transcriptional regulator CcpA (Carbon Control Protein A) coordinately controls a broad cross-section of the *S*. *pyogenes* transcriptome including both virulence and metabolic pathways [19–21]. Analysis of mutations that inactivate the ability of CcpA to bind DNA vs those that lock it into its high-affinity DNA-binding conformation revealed that both types of mutations lead to attenuation of tissue damage [22]. However, inactivating mutations result in attenuation due to a reduction in bacterial growth and accumulated tissue burden. In contrast, locked-on mutations have uncoupled the growth/damage relationship resulting in wild-type levels of bacterial tissue burdens in the absence of tissue damage [22]. Since CcpA’s principal function is carbon catabolite repression, acting to adjust metabolism in response to the availability of preferred growth substrates [23], these data show that *S*. *pyogenes* contains systems that can couple metabolic signals with regulation of the virulence transcriptome to dynamically balance growth rates against tissue damage.

While these studies establish a relationship between the virulence transcriptome and metabolism, how the flow of carbon itself through the alternative pathways for pyruvate reduction contributes to pathogenesis remains unclear. In the present study, we employ a genetic approach to interrogate how carbon flow impacts growth/damage balance and the influence of aerobic vs. anaerobic metabolism in the lactic acid pathogen, *Streptococcus pyogenes*. Mutants were constructed to inactivate key enzymes that control the entry of carbon into each of the alternative pathways for reduction of pyruvate in order to constrain metabolic plasticity. Virulence was then assessed in a murine model of soft tissue disease that allows for the independent assessment of growth in tissue vs. the degree of tissue damage and through infection of macrophages *in vitro* to assess the impact of *S*. *pyogenes* metabolism on immunoregulation. This analysis revealed a key role for mixed acid fermentation, the environmental availability of oxygen, macrophages, and the regulatory cytokine IL-10 as important regulators of the *S*. *pyogenes* growth/damage balance.

## Results

### Three alternative pathways for pyruvate reduction

**Fig 1:**
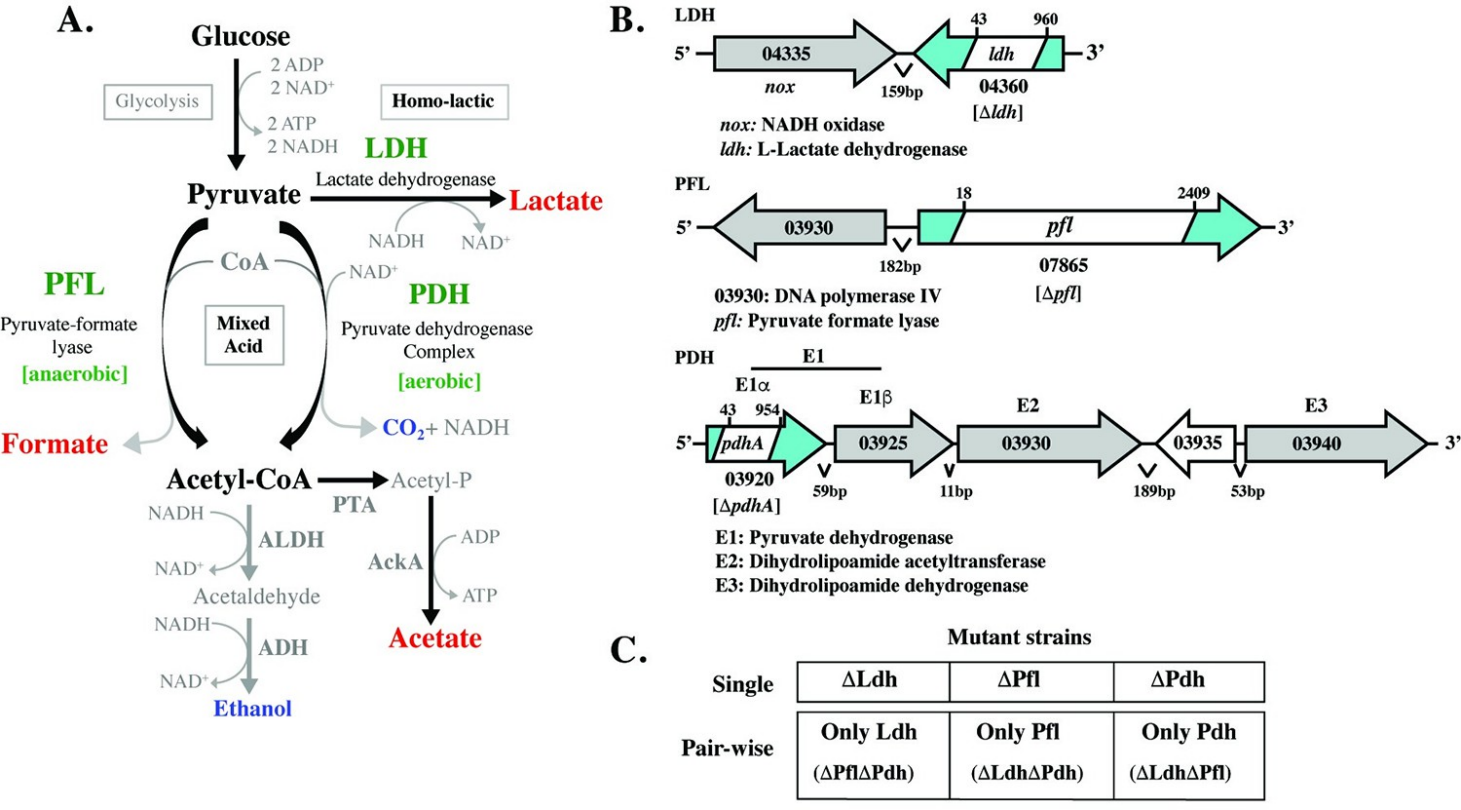
*S*. *pyogenes* Central Carbon Metabolism and Mutant Construction. **A. Fermentation pathways**. Homo-lactic and mixed-acid fermentation pathways are shown. Major short chain fatty acid end-products are shown in red, other end-products are shown in blue and the enzymes acting on pyruvate are shown in green. Other enzymes listed: ADH, alcohol dehydrogenase; PTA, phosphate acetyltransferase; AckA, acetate kinase. **B. Enzyme loci**. The chromosomal regions encoding the indicated enzymes are shown, with the annotation and genetic loci listed corresponding the genome of *S*. *pyogenes* HSC5 [27]. Enzyme open-reading frames are shown in blue with the location of the constructed in-frame deletions shown in white. Deletion junctions are indicated by “x” and “y” relative to the first base-pair of the open-reading frame. **C**. **Mutant strain nomenclature**. The indicated nomenclature was adopted for clarity. Each cell shown represents a mutant strain. For single mutants, the name corresponds to the genotype. For double mutants, the name corresponds to the enzyme that remains active. Mutant genotypes are in parentheses. Specific strain names and genotypes are provided in **S1 Table**.

Classically considered a lactic acid bacterium, *S*. *pyogenes* can reduce pyruvate to lactate via homo-lactic fermentation using the enzyme lactate dehydrogenase (**LDH**) through oxidation of NADH to NAD^+^ to balance the accumulation of NADH generated during glycolysis (**Fig 1A**). However, like most lactic acid bacteria, *S*. *pyogenes* possesses metabolic plasticity and can reduce pyruvate via mixed-acid fermentation, which consists of a collection of pathways that oxidize NADH through generation of alternative short chain fatty acid (**SCFA**s) end-products (formate, acetate) and critical metabolic intermediates, including acetyl-CoA [17] (**Fig 1A**). For *S*. *pyogenes*, mixed-acid fermentation consists of two parallel arms that are defined by the major enzyme complexes that act on pyruvate. These include pyruvate-formate lyase (**PFL**) and the pyruvate dehydrogenase complex (**PDH**) (**Fig 1A**). It is generally considered that PFL only functions under anaerobic conditions, while PDH is only active in aerobic environments [24–26]. The net result of both arms is the production acetyl-CoA, although each generates different end-products (**Fig 1A**). Further metabolism of acetyl-CoA oxidizes NADH and produces additional ATP (**Fig 1A**). Under aerobic conditions the homo-lactic and mixed-acid pathways can have a hierarchical relationship, as once glucose is depleted, the lactate generated can be recovered to pyruvate by the consumption of oxygen by the enzyme lactate oxidase (**LctO**). Pyruvate is then metabolized by PDH with the net result being the generation of acetyl-CoA, an additional end-product, H_2_O_2_, and additional ATP [19] (**S1 Fig**).

In order to unravel the relationship between these pathways and their potential roles in the regulation of growth/damage balance, our strategy was to restrict carbon flux by constructing non-polar in-frame deletions in each of the three major enzymes that act on pyruvate, both singly and in each pair-wise combination. These included mutations in LDH (genomic locus L897_04360), PFL (L897_07865) and PDH subunit E1⍺ (L897_03920) [27] (**Fig 1B**). The wild type (WT) host for these studies was *S*. *pyogenes* strain HSC5, whose phenotype in several murine models of infection is well-characterized [22,28,29]. The resulting single and pair-wise mutant strains and their respective genotypes are listed in **Fig 1C** and **S1 Table.**

### Distinct fermentation pathways are required for growth in aerobic vs. anaerobic environments

**Fig 2:**
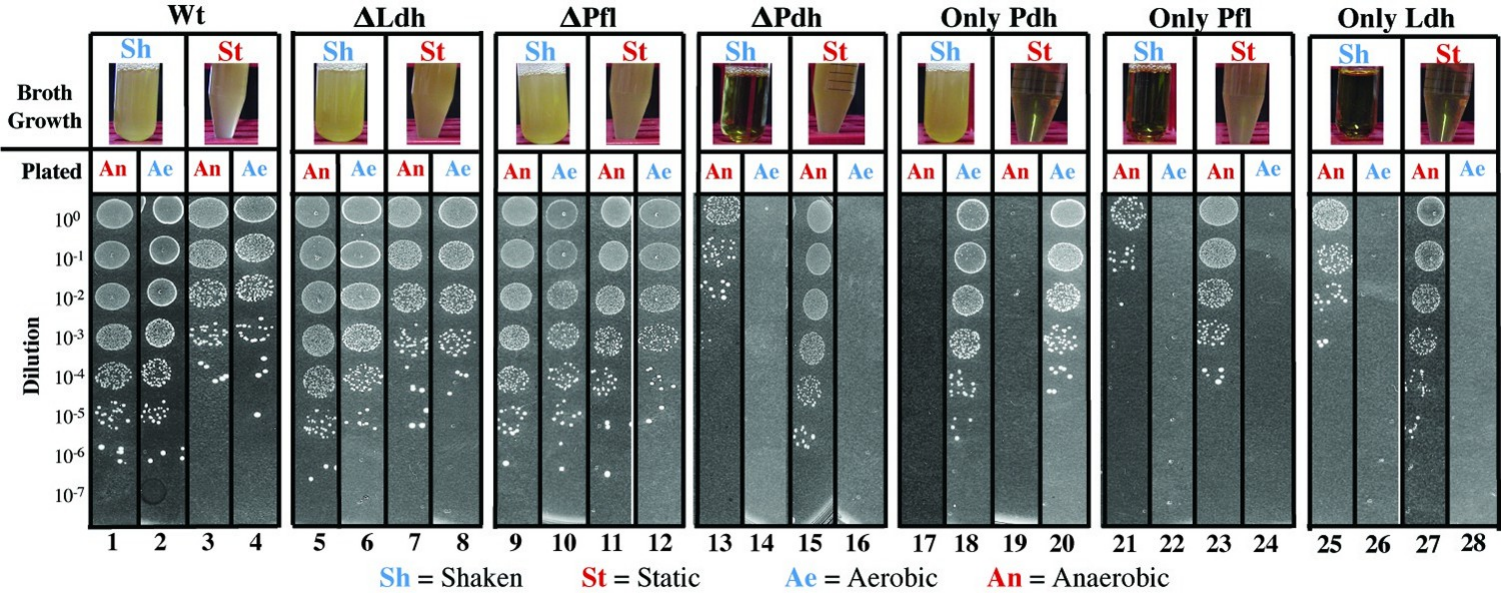
Growth characteristics under ideal conditions. Shown is the ability of the various mutants listed at the top of the Figure to grow in a standard nutrient rich medium (THY medium) cultured under oxygenated (Shaken, aerobic) or oxygen-limited (Static, anaerobic) conditions, as listed. The top panel (**Broth Grown**) presents images of culture tubes follow overnight culture in liquid THY medium. The degree of turbidity corresponds to the amount of growth, which was quantitated by spot plating on solid media (THY plates) as shown in the bottom panel (**Plated**) under the atmospheric conditions indicated. The spot plate data presented are a montage assembled from individual images collected from the same experiment, that are delineated by the boxes numbered at the bottom of the Figure. Culture conditions are described in detail in the **Materials and Methods**. Strains are as described in Fig 1.

To evaluate the role of these pathways under different atmospheric conditions, the growth characteristics of the mutant panel (**Fig 1C**) was analyzed using a standard nutrient rich medium (Todd Hewitt plus 1% yeast extract) in the presence or absence of oxygen. Cultures were grown overnight in liquid media under shaken (Sh, aerobic) or static (St, oxygen-limited) conditions (Broth Growth, **Fig 2**) and then plated on solid media to determine colony forming units (CFUs) under anaerobic (An) or aerobic (Ae) conditions (Plated, **Fig 2**. See “Materials and Methods”). In this assay, the WT strain grew well regardless of whether the inoculum was cultured under static or shaken conditions, although higher numbers of CFUs were obtained under aerobic conditions (compare panels 1, 2 vs. 3, 4; **Fig 2)**. Single mutants lacking LDH or PFL grew as well as, or better than WT (compare ΔLdh and ΔPfl to WT, **Fig 2**). However, the single mutant lacking PDH lost its ability to grow aerobically (ΔPdh, **Fig 2**), while the mutant solely dependent on PDH could only grow under aerobic, but not anaerobic conditions (Only Pdh, **Fig 2**). In contrast, the double mutants lacking PDH (Only Pfl, Only Ldh) could grow anaerobically, but not aerobically (**Fig 2**). Taken together, these data demonstrate that PDH is absolutely required for aerobic growth, but cannot support anaerobic growth, while PFL and LDH are at least partially redundant for growth under anaerobic conditions.

### SCFA end-product profile and growth over time are tunable depending on fermentation pathway and oxygen exposure

**Fig 3:**
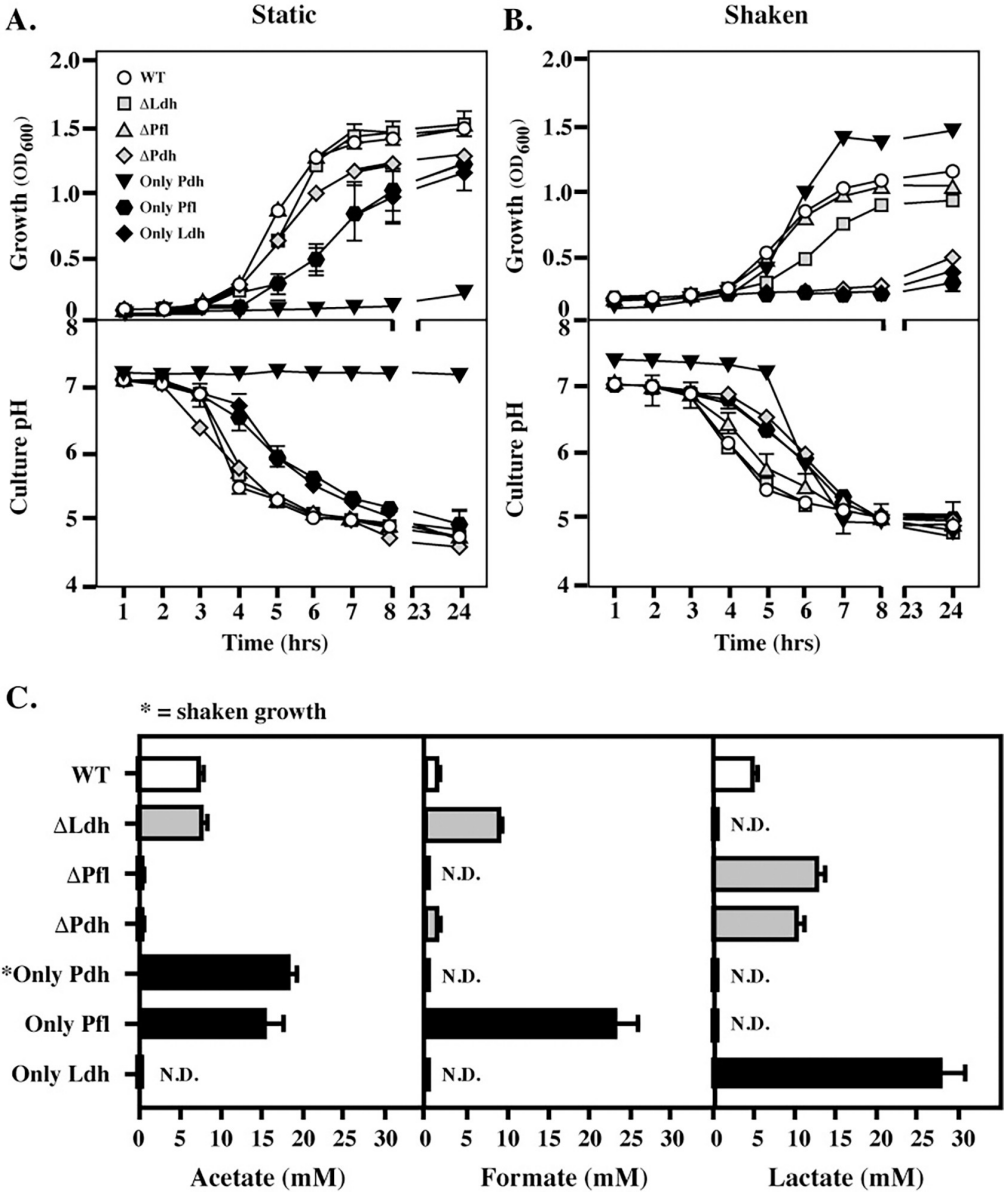
Growth characteristics under conditions reproducing the soft tissue environment. Comparison of the growth and changes in culture pH for the mutant panel was analyzed in a medium that reproduces conditions encountered by the bacterium during soft tissue infection (C medium + 0.1% glucose) under **(A)** oxygen-limited (Static) and **(B)** oxygenated (Shaken) conditions, with (**C**) the concentrations of the 3 major SCFA end-products determined following oxygen-limited growth. An exception is Only Pdh, whose SCFA profile was determined following culture in Shaken conditions since this mutant cannot grow under oxygen limited conditions. N.D. indicates levels were below the limit of detection. Data presented represents the mean and standard error of the mean derived from three experiments.

The contribution of these pathways to metabolic plasticity was next evaluated using an *in vitro* medium (C medium + 0.2% glucose) which has been shown to reproduce the behavior of the transcriptome in a murine subcutaneous ulcer model of soft tissue infection [15,16,30]. Culture density (OD_600_) and culture pH were monitored over 24 hrs to assess bacterial growth and metabolic activity, respectively. Similar to rich medium, growth with the loss of LDH or PFL under static conditions did not differ from WT (ΔLdh, ΔPfl vs. WT, **Fig 3A**). Double mutants dependent solely on LDH or PFL could also grow, although at reduced rates and yields (Only Ldh, Only Pfl; **Fig 3A**), which confirms that these pathways are partially redundant. In contrast, the double mutant dependent on PDH could not grow or acidify media in static culture (Only Pdh, **Fig 3A;** top and bottom), while the other mutants could acidify media at rates consistent with their growth patterns (**Fig 3A,** bottom). Under aerobic (Shaken) conditions, all single or double mutants that lacked PDH could not grow (ΔPdh, Only Ldh, Only Pfl; **Fig 3B** top), while all strains with PDH could grow (WT, ΔLdh, ΔPfl; **Fig 3B** top) and the mutant solely dependent on PDH grew at a higher rate and yield compared to WT (Only Pdh, **Fig 3B**, top), consistent with an ability of this pathway to make additional ATP (**Fig 1A**). Of note, all strains demonstrated metabolic activity as indicated by acidification of the media, even when they were not able to grow (**Fig 3B**, bottom). Taken together, these data show PDH is essential for aerobic growth and cannot support anaerobic growth, while LDH and PFL are partially redundant for anaerobic growth.

### Carbon flow and growth in pathway constrained mutants

The SCFA end-products of *S*. *pyogenes* fermentation, including lactate, formate and acetate (**Fig 1**), have potent immunoregulatory properties [7,8]. To understand how end-product profiles are impacted by each enzyme, the concentrations of acetate, formate and lactate were quantitated during culture in C medium + 0.2% glucose under static conditions, which supported the growth of all mutants except Only Pdh, which was analyzed following growth in shaken cultures (**Fig 3A and 3B**). SCFA concentrations were determined at 24 hrs in culture supernatants and normalized for growth. Under these conditions, WT produced a mixture of all three SCFAs (**Fig 3C**). As expected, mutants lacking Ldh did not produce lactate and the mutant solely dependent on Ldh (Only Ldh) produced lactate exclusively at levels approx. 5 times higher than WT (**Fig 3C**). Lactate was also the major end-product of either of the single mutants in the alternative mixed-acid pathways (ΔPfl, ΔPdh; **Fig 3C**). For mutants solely dependent on a single arm of mixed-acid fermentation, Only Pfl produced a mixture of acetate and formate, while Only Pdh exclusively produced acetate (**Fig 3C**). This may explain its enhanced growth yields under this condition (see **Fig 3B**), as production of acetate yields additional ATP (see **Fig 1A**).

### Mixed-acid, but not homo-lactic fermentation influences growth/damage balance

**Fig 4:**
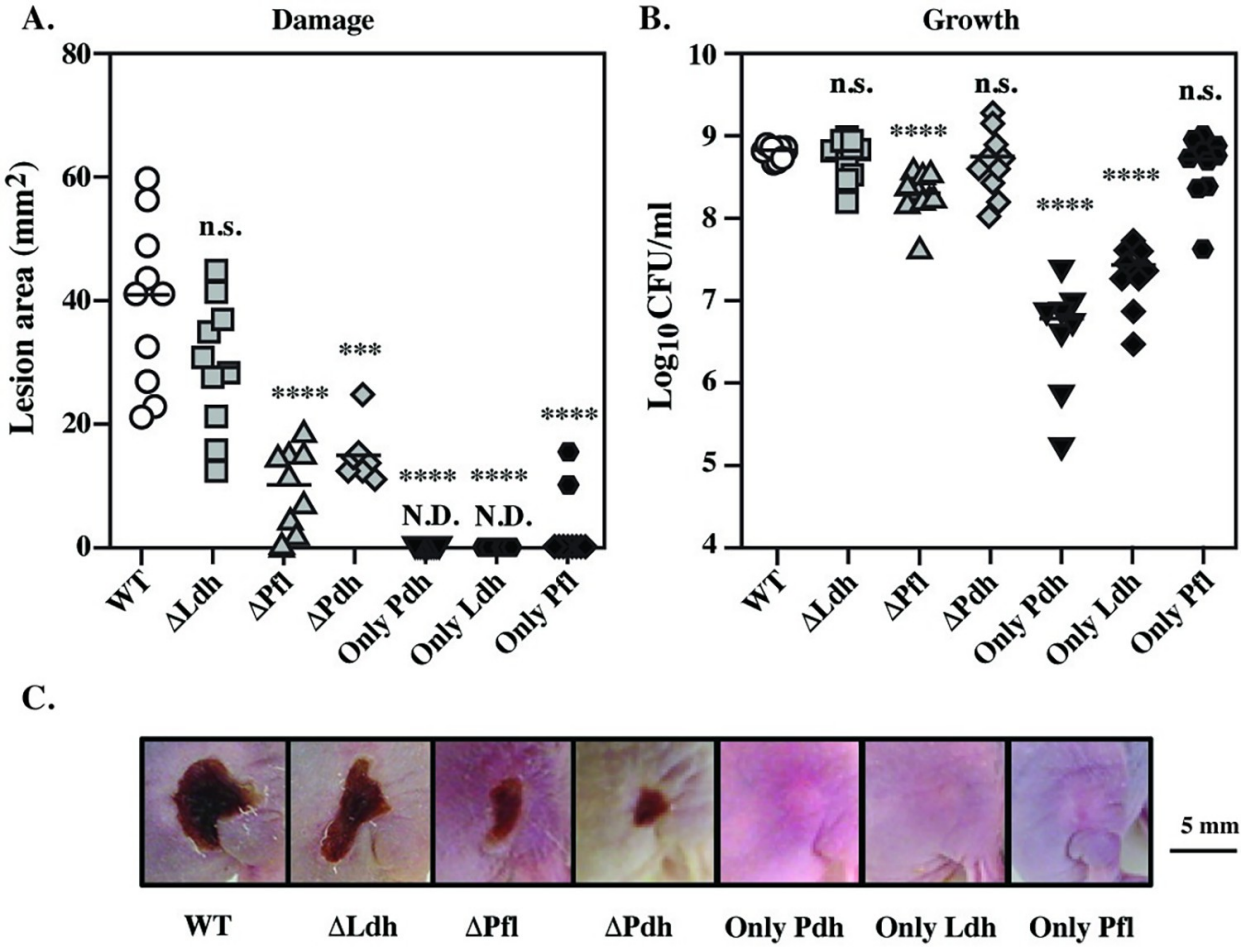
Non-overlapping roles for mixed acid fermentation in soft-tissue infection. A subcutaneous ulcer model of soft-tissue infection of SKH1-E mice was used to compare virulence properties between mutant and WT, which allows the determination of **(A)** tissue damage, measured as the area of the draining ulcer and **(B)** growth in tissue, measured as the number of recoverable CFUs. Data presented were collected at Day 3, with **(C)** showing images from representative Day 3 ulcers. For A and B, each symbol represents an individual mouse, pooled from two individual experiments, with differences compared to WT. ***, *P* < 0.05; ****, *P* < 0.001; n.s., not significant. N.D. indicates a visible ulcer was not detected.

The contribution of carbon flow through these fermentative pathways was analyzed for their role in pathogenesis using a murine model of soft tissue infection that allows independent assessment of growth in tissue vs. tissue damage [28,29,31]. In this model, immunocompetent SKH1-E hairless mice are injected subcutaneously with 10^7^ CFU of streptococci, which, for the WT strain (HSC5), results in a local lesion that becomes a necrotic ulcer by 24 hrs. The ulcer produces an eschar and enlarges over the course of 3 days, followed by a period of healing and the infection does not progress to systemic disease [28]. Metrics for comparison of strains include assessment of tissue damage by measurement of lesion area and assessment of growth by determination of CFU tissue burdens at the time of peak lesion formation by the WT strain (3 days post-infection). Surprisingly, *S*. *pyogenes*, a canonical lactic acid bacterium, showed neither a growth nor damage defect in the absence of homo-lactic fermentation (ΔLdh), as indicated by no significant reduction in lesion area or tissue burden when compared to WT (**Fig 4A–4C**). Conversely, all mixed acid pathway mutants had significantly reduced tissue damage (**Fig 4A and 4C**) with the PFL and PDH pathways each having unique effects on growth/damage balance. For all strains lacking PFL (ΔPfl, Only Ldh, Only Pdh), reduction in tissue damage was correlated with reduced growth in tissue. The double mutants lacking PFL (Only Pdh, Only Ldh) had the greatest decrease in tissue burdens with an average of 1.5–2.5 log reduction in recovered CFU (**Fig 4**). This growth defect was reversed in all strains that contained PFL (ΔPdh, ΔLdh, Only Pfl), indicating PFL is essential for growth in soft tissue infections.

In contrast, strains lacking PDH had a strikingly different presentation. A reduction in tissue damage did not always correlate with a reduction in tissue burden. Mutants ΔPdh and Only Pfl had an uncoupled growth/damage balance, with a significant reduction in their ability to damage tissue (**Fig 4A and 4C**), while exhibiting no significant reduction in their ability to grow in tissue (**Fig 4B**). An exception was Only Ldh, which was attenuated for both damage and growth (**Fig 4A–4C**). Taken together, these data show that: **i.)** Mixed-acid, but not homo-lactic fermentation is required for pathogenesis in soft tissue, **ii.)** Both the anaerobic (PFL) and aerobic (PDH) arms of mixed-acid fermentation contribute to soft tissue pathogenesis, but by different mechanisms, **iii.)** PFL is required for growth in soft tissue, and **iv.)** fermentation via PDH plays a key role in modulating the growth/damage balance (**Table 1**).

**Table 1:** Growth/Damage Phenotypes.

	Compared to WT^1^		
Mutant	Growth	Damage	Phenotype^2^
ΔLdh	+	+	Not Attenuated
ΔPfl	-	-	Attenuated
ΔPdh	+	-	Altered
Only Pdh	-	-	Attenuated
Only Ldh	-	-	Attenuated
Only Pfl	+	-	Altered

^1^[(+), not significantly different; (-), significantly different] vs. WT. See Fig 4.

^2^(+/+), Not Attenuated; (-/-), Attenuated; (+/-) or (-/+), Altered.

### PDH controls macrophage phagosome acidification

**Fig 5:**
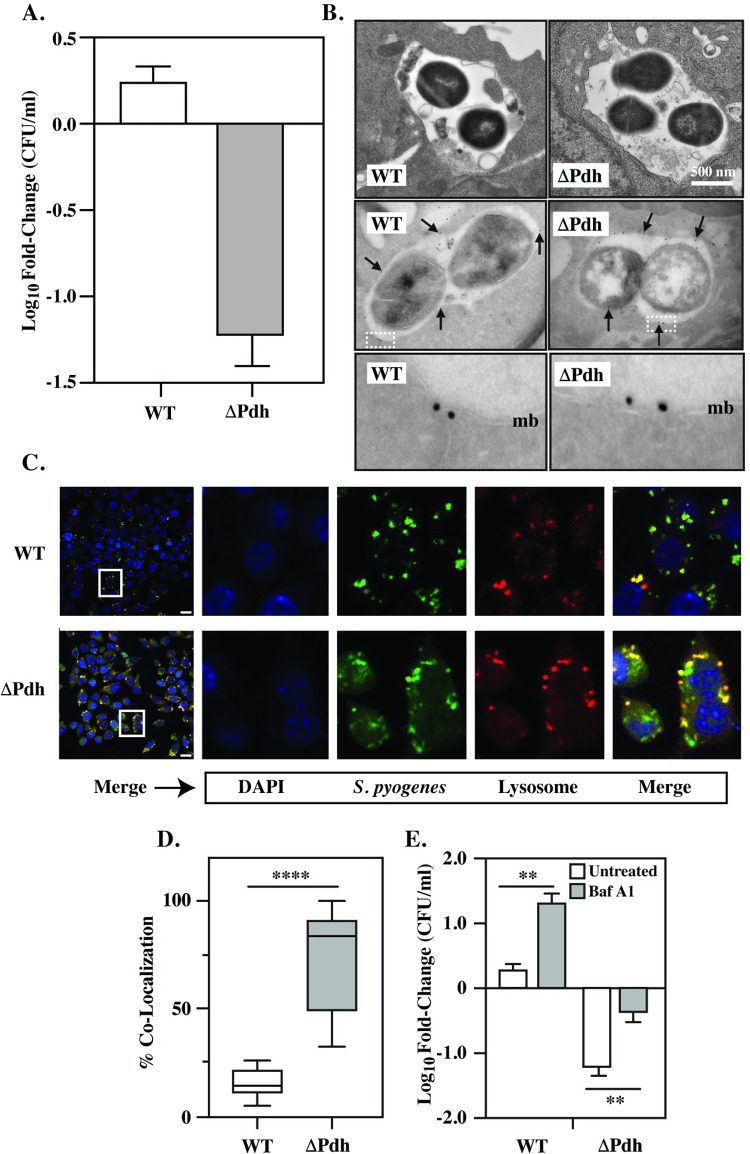
Comparison of macrophage infection by WT and ΔPdh. Cultured Raw264.7 macrophages were infected by the indicated strains as detailed in the Materials and Methods. **A.** Net change in CFUs recovered between time 0 and 4 hrs of infection. **B.** Transmission electron microscopy of infected Raw264.7 cells (top), with immunogold staining to detect Lamp1 (middle) Arrows indicate several representative gold particles. The bottom panel shows the regions outlined by the boxed areas in the middle panels at 7.5X magnification. mb, vacuolar membrane. **C.** Fluorescent microscopy of infected Raw264.7 cells to detect DNA (DAPI), *S*. *pyogenes* (Cell Tracker Green) and Lysosomes (LysoTracker Red). Merge overlays all three stains. Images of the 4 panels at the right are magnified from the area indicated by the boxes shown in the merged images at the left. **D**. Quantitation of the number of bacteria which co-localized with lysosomes. The top and bottom of each box delineates the 75^th^ and 25^th^ percentiles, respectively; the horizontal bar indicates the median. **E**. Net change in recoverable CFUs between untreated Raw264.7 cells, and cells treated with Bafilomycin A1 (Baf A1). Where indicated, data presented represents the mean and standard error of the mean derived from at least 3 independent experiments and examination of at least 1,000 cells. **, *P* < 0.01; ***, *P* < 0.05; ****, *P* < 0.001.

To pursue how aerobic mixed acid fermentation regulates the growth/damage balance, we focused on ΔPdh, the single mutant lacking PDH. Complementation of ΔPdh restored WT profiles of growth during *in vitro* culture (**S2A and S2B Fig**) and for growth/damage balance during infection (**S2C and S2D Fig**). As resident and monocyte-derived macrophages regulate cutaneous immune responses and since monocyte-derived macrophages play a critical immunoregulatory role in the development of subcutaneous ulcers in the murine model [32], macrophages were evaluated as possible influencers of the growth/damage balance. To test this, cultured Raw264.7 macrophages or bone marrow-derived macrophages (BMDM) were infected with ΔPdh or the WT strain. After 4 hrs of infection, intracellular survival was compared, revealing a significant difference in recoverable CFUs. Contrary to equivalent tissue burdens between WT and ΔPdh infected mice, CFUs recovered for ΔPdh decreased by >1 log vs. time 0 hrs, whereas CFUs for WT slightly increased for Raw264.7 cells (**Fig 5A**). Similar results were obtained with BMDM (**S3A Fig**). At this time point, the viability of Raw264.7 macrophages infected by either strain was >95% (**S4 Fig**). Complementation of ΔPdh restored WT levels of growth in Raw264.7 cells (**S2E Fig**). Consistent with prior reports [33–35] both WT and ΔPdh were located in membrane-bound vacuoles positive for the lysosome-associated protein, Lamp1, as determined by immunogold electron microscopy of Raw264.7 cells **(Fig 5B**), indicating that this phagosomal compartment has fused with lysosomes. Also consistent with prior reports, WT prevented acidification of this compartment, despite fusion with lysosomes (**Fig 5C and 5D**). In contrast, almost all vacuoles containing ΔPdh were acidic (**Fig 5C and 5D**), showing that the mutant cannot prevent acidification. Blocking acidification by treatment with the vacuolar H^+^ ATPase inhibitor Bafilomycin A1 at a non-cytotoxic concentration [36] significantly enhanced the survival of both ΔPdh and WT (**Fig 5E**), consistent with the observation that the viability of both WT and ΔPdh decreased when exposed to an acid shock *in vitro* (1 hr exposure to pH 4.5), with ΔPdh being significantly more sensitive than WT (**S5A Fig**). An inability to resist acid stress likely makes a bigger contribution to ΔPdh’s reduced survival in macrophages than oxidative stress, as buffering media rescues higher growth yields than scavenging peroxide under *in vitro* aerobic growth conditions (**S5B and S5C Fig**). These data indicate that: **i.)** Acidification of the phagolysosome limits the ability of *S*. *pyogenes* to persist with in macrophages, **ii.)** PDH makes an important contribution to the ability of *S*. *pyogenes* to block acidification; and **iii.**) Its sensitivity to phagolysosome acidification suggests that survival within macrophages cannot account for high tissue burdens observed in ΔPdh infected mice.

### PDH controls IL-10/TNFα balance

**Fig 6:**
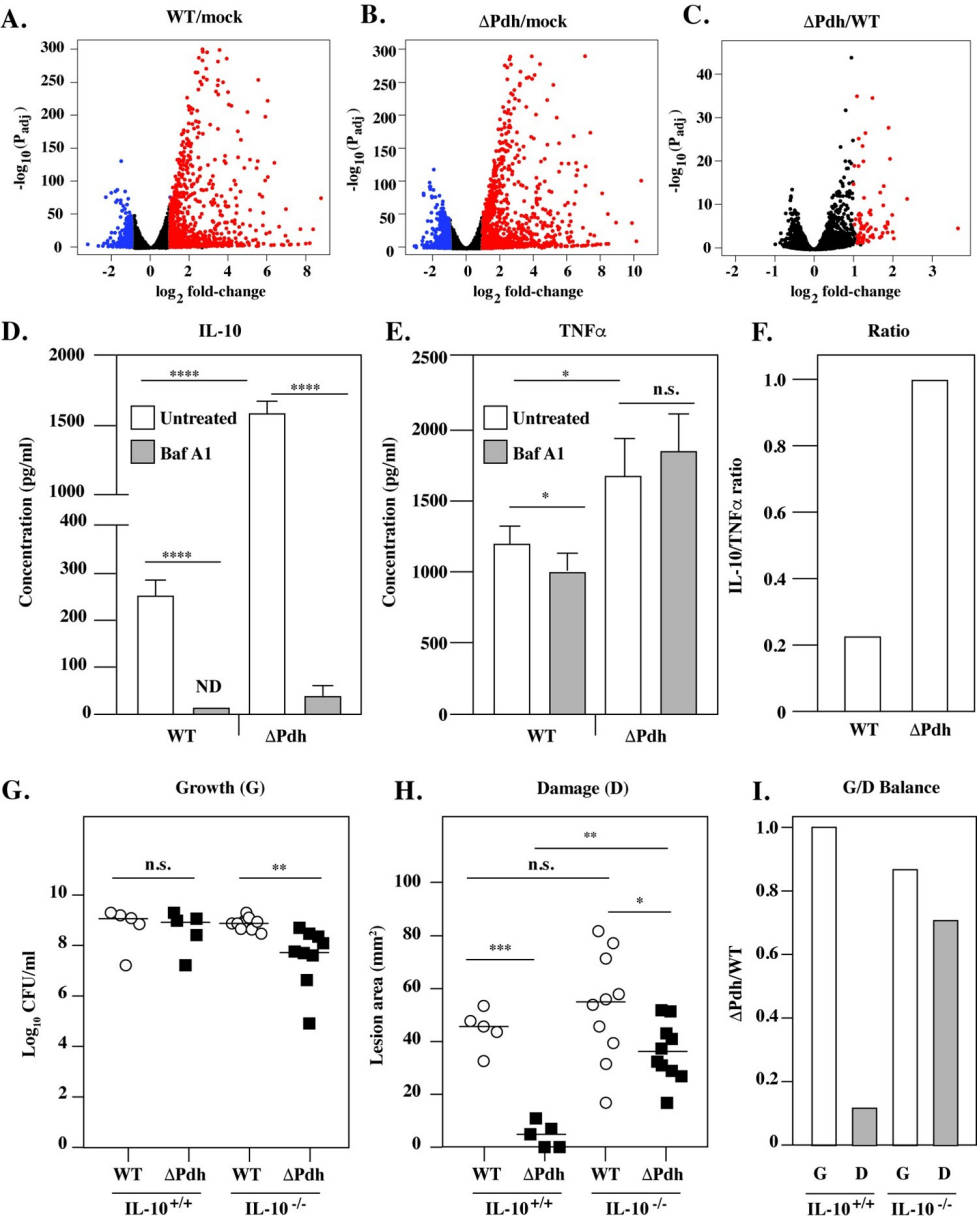
Differential expression of IL-10 between WT- and ΔPdh-infected macrophages. Cultured Raw264.7 cells were infected by WT, ΔPdh or PBS (mock) for 4 hrs. Overall differences in gene expression are shown in Volcano plots of statistically significantly differentially expressed genes for the indicated comparisons (**A, B, C**). Genes regulated up or down regulated with a log_2_ fold-change > 1.0 are shown in red and blue, respectively. Supernatants from cells infected and untreated or treated with Bafilomycin A1 (Baf A1) were analyzed to determine concentrations of IL-10 (**D**) and TNFα (**E**) and the IL-10/TNFα ratio determined for untreated infected cells (**F**). Subcutaneous infection of the indicated strains in IL-10^+/+^ and IL-10^-/-^ C57BL/6 mice is shown, comparing Growth (**G**), Damage (**H**) and the Growth/Damage ratio calculated for ΔPdh vs WT (**I**). For G and H, each symbol represents an individual mouse, pooled from two individual experiments, with differences compared to WT. Where indicated, *, *P* < 0.05; **, *P* < 0.05; ****, *P* < 0.001; n.s., not significant.

Since numerous immunomodulatory receptors reside within the vacuole that may differentially signal macrophage response pathways, an RNA-seq analysis was conducted to compare differential changes in the transcriptome in response to infection by WT vs. ΔPdh. When infection with either strain was compared to mock-infected Raw264.7 cells, a similar profile was observed, with 1025 genes up-regulated and 175 genes down-regulated (**Fig 6A and 6B**). A computational pathway enrichment analysis using Reactome [37] indicated that the majority of these differentially regulated genes are involved in immunoregulation (not shown). Comparison of ΔPdh to WT infected macrophages revealed that no genes were significantly down-regulated, but that 78 genes were up-regulated (**Fig 6C and S2 Table**). Pathway enrichment analysis placed these genes predominantly in cytokine signaling pathways, with the IL-10 pathway as the most significantly up-regulated by ΔPdh (*P* = 1.1 x 10^−16^, **S3 Table**). Due to its potent anti-inflammatory properties [38], the ratio of IL-10 to the pro-inflammatory cytokine TNFα is often used as a measure of inflammatory homeostasis [39]. Determination of this ratio for infected Raw264.7 cells revealed a >6-fold increase in IL-10 expression when compared to WT (**Fig 6D**). In contrast, TNFα levels were increased only about 1.3-fold by ΔPdh (**Fig 6E**). Complementation of ΔPdh restored a WT profile for expression of IL-10 and TNFα (**S2F and S2G Fig**). Blocking acidification with Bafilomycin A1 completely inhibited expression of IL-10, while a similar treatment had essential no effect on expression of TNFα (**Fig 6D and 6E**). Overall, infection by ΔPdh resulted in a 5-fold increase in the IL-10/TNFα ratio (**Fig 6F**). These results were not unique for Raw264.7 cells, as equivalent results were obtained from BMDM for bacterial survival, expression of IL-10 and TNFα, and the IL-10/TNFα ratio (**S3B–S3D Fig**). These data establish: **i.)** That phagolysosomal acidification is essential for expression of IL-10, but not TNFα, and **ii.)** That aerobic mixed acid fermentation regulates the inflammatory signaling balance in macrophages through manipulation of IL-10 expression.

### IL-10 is required for PDH-mediated control of growth/damage balance

Differential secretion of IL-10 in macrophages *in vitro* suggests that IL-10 may be a key factor for modulation of growth/damage balance during *S*. *pyogenes* soft-tissue infections. This model predicts that in the absence of IL-10, infection by WT and ΔPdh will produce a similar growth/damage balance. To test this, we compared WT and ΔPdh infection of IL-10^+/+^ and IL-10^-/-^ C57BL/6 mice. Similar to infection of SKH1-E mice (see above), ΔPdh grew to levels similar to WT during infection of IL-10^+/+^ mice (**Fig 6G**), while producing significantly less damage to tissue (**Fig 6H**), resulting in an altered growth/damage balance (IL-10^+/+^, **Fig 6I**). However, while there was a slight decrease in growth for ΔPdh as compared to WT in IL-10^-/-^ mice (**Fig 6G**), there was a >5-fold increase in its ability to damage tissue (**Fig 6H**), which resulted in a growth/damage balance similar to that of WT (**Fig 6I**). These data establish that *S*. *pyogenes* can inhibit IL-10 expression by a mechanism dependent on its pathway of aerobic mixed acid fermentation and that inhibition of PDH activity may provide new therapeutic strategies to limit tissue damage during severe invasive *S*. *pyogenes* infections.

## Discussion

It is becoming increasingly appreciated that alternative fermentative pathways for the reduction of pyruvate play a pivotal role in the commensal host-bacteria relationship through the generation of immunomodulatory metabolic end products [7,8,40]. However, the relative contributions that these pathways make to the pathogenic host-bacteria relationship is not well-understood. In the present study, we have used a genetic approach to comprehensively investigate the relative contributions of alternative fermentative pathways to the pathogenesis of a canonical lactic acid bacterium. When examined in the context of *S*. *pyogenes* soft-tissue infection, the data show that its pathway for homolactic fermentation makes only a minor contribution to virulence. Instead, pathways for mixed-acid fermentation play critical and non-overlapping roles. The anaerobic arm of mixed-acid fermentation (PFL) is required for growth in soft tissue, while the aerobic arm (PDH) influences growth/damage balance through its ability to manipulate host cytokine responses. The streamlined metabolic potential of *S*. *pyogenes* suggests that therapeutic manipulation of key fermentative enzymes may represent a novel approach for altering growth/damage balance to improve treatment outcomes for severe tissue destructive *S*. *pyogenes* diseases.

**Fig 7:**
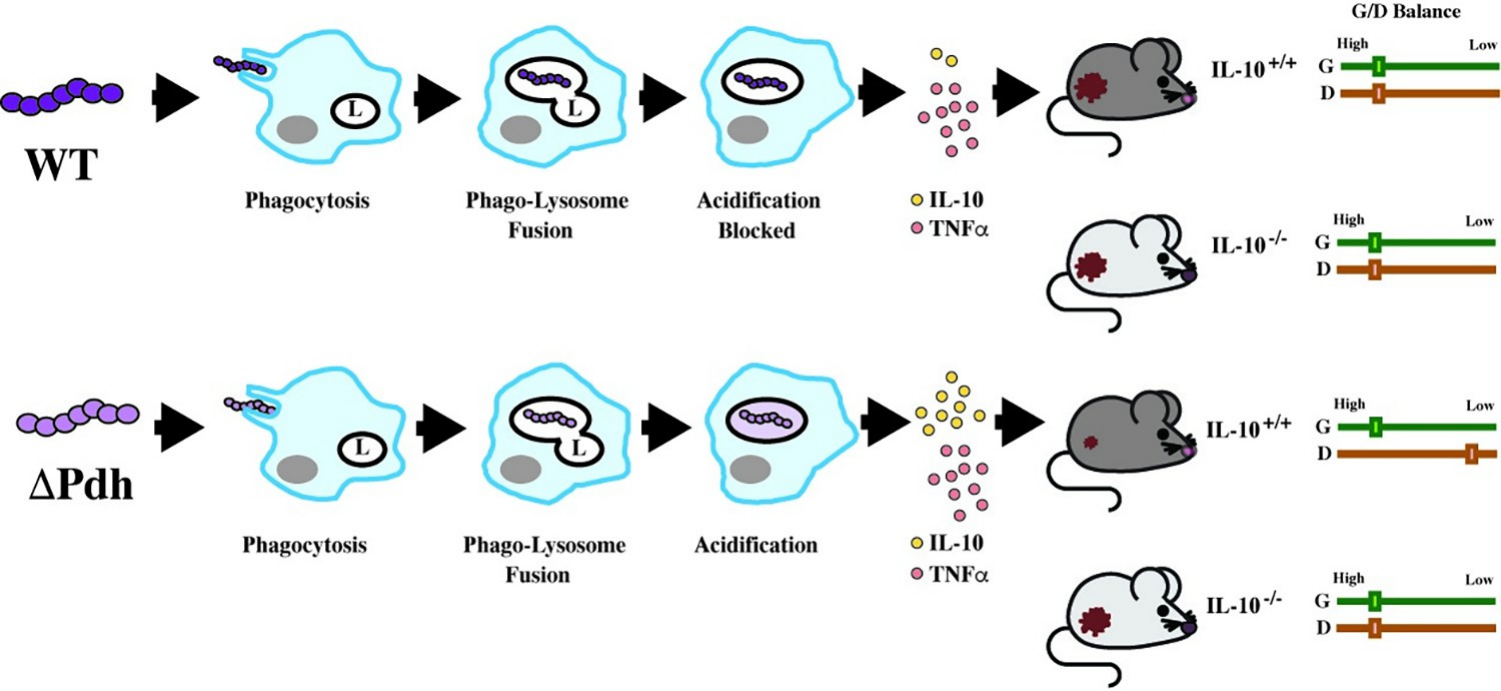
Model for PDH regulation of growth/damage balance. Shown is a comparison of WT and ΔPdh mutant phenotypes observed in macrophages *in vitro* and for murine subcutaneous infection. The steps of infection in macrophages are shown in temporal order from left to right by the closed arrows and are labeled as indicated (featuring: L, lysosome; grey oval, nucleus), resulting in the ratio of the cytokines IL-10 and TNFα represented by frequency of the symbols indicated, which correlates (as shown by the open arrows) to the mouse infection phenotypes shown at the right of the Figure. Relative areas of the resulting lesions are represented as small or large in mice whose IL-10 genotypes are noted. Growth/Damage balance (G/D Balance) for IL-10^+/+^ or IL-10^-/-^ mice are shown by the paired sliders at the very right of the Figure, with the relative degree of Growth (G, green) and Damage (D, red) indicated by a box on a scale from Low to High, as shown.

Our comparison of WT and ΔPdh, using *in vivo* and *in vitro* models, supports the following model for cutaneous infection in the murine model (**Fig 7**): WT and ΔPdh are phagocytosed, followed by fusion of the phagocytic vesicle with lysosomes. However, while WT can block the acidification of the phagolysosome [33], ΔPdh has lost this ability, leading to the production of considerably more IL-10. Since levels of TNFα do not change, the inflammatory index is altered such that higher IL-10 levels correlate with an altered growth/damage balance favoring a reduction in tissue damage (**Fig 7**). These data suggest that WT *S*. *pyogenes* actively manipulates macrophage cytokine signaling to suppress the expression of IL-10. Consistent with this, the loss of IL-10 in IL-10^-/-^ mice does not alter growth/damage balance for infection by the WT strain, but does for infection by ΔPdh (**Fig 7**). More comprehensive studies on cytokine expression *in vivo*, phagolysosomal maturation and the role of macrophages *in vivo* will be required to confirm this model. However, these data show that **i**.) *S*. *pyogenes* can manipulate growth/damage balance in soft tissue, **ii**.) that the mechanism depends on the aerobic arm of mixed-acid fermentation, **iii.**) that the target of this response is IL-10; and **iv.**) that macrophages may be the effector cell of this response.

Macrophages represent an ideal target for *S*. *pyogenes* to manipulate growth/damage balance as monocyte-derived macrophages recruited from the circulation play a critical regulatory role in formation of *S*. *pyogenes* ulcers [32,41]. Recruited monocyte-derived macrophages also regulate the wound healing response where control of the balance of pro- vs. anti-inflammatory cytokines plays an essential role (reviewed by [42]). Temporal management of cytokine balance is accomplished largely by kinetic regulation of macrophage differentiation into the M1-like pro-inflammatory vs. the M2-like anti-inflammatory phenotypes, the latter which produces high levels of IL-10 [42]. An altered temporal pattern of differentiation disrupts wound healing, often resulting in chronic non-healing wounds [42]. The role of macrophage differentiation in the acute and healing stages of subcutaneous infection and whether this is manipulated by the aerobic metabolism of *S*. *pyogenes* remains to be determined. Our data suggests pathway enrichment analysis of genes differentially regulated in macrophages by ΔPdh identified the IL-4/IL-13 pathway (**S3 Table**), which plays an important role in the polarization of macrophages to the M2-like phenotype [43]. It is known that polarization and activation of antimicrobial function in macrophages can be influenced by SCFAs [44], suggesting that SCFA production from *S*. *pyogenes* metabolism, including the altered SCFA pattern of the ΔPdh mutant, directs transcriptional changes in macrophages leading to changes in cytokine production.

Regulation of tissue damage represents an intersection between the pathogen’s growth/damage response and the host’s disease tolerance response. While the former is directed by the pathogen through regulation of virulence factor expression, the latter is a host response which acts to limit collateral damage to tissue caused by the immune response, often by up-regulation of host cell functions that resist and repair damage [45,46]. The present study suggests that the intersection between these two responses centers on PDH and a previously unappreciated aerobic niche. If this niche resides in macrophages, our data indicate at least two roles for PDH: **i**) blocking phagolysosomal acidification and **ii.**) supporting resistance to acid stress. For the former, it has been shown that *S*. *pyogenes’* ability to block phagolysosomal acidification requires the secreted toxins Streptolysin O (SLO), the NAD glycohydrolase and a unique toxin delivery pathway known as Cytolysin-Mediated Translocation [33,47,48], suggesting that similar to many other *S*. *pyogenes* virulence factors, expression of these toxins is linked to central carbon metabolism [12]. However, deletion of SLO does not result in increased IL-10 expression by infected macrophages *in vitro* (**S6 Fig**), suggesting that PDH is also required to resist acid stress. PDH contributes to the production of Acetyl-CoA (see **Fig 1**), that in other streptococcal species is essential for lipid synthesis and membrane remodeling for adaptation to acid stress [49]. A deficient acid stress response may explain the reduced viability of ΔPdh in the phagolysosome that can be rescued by chemical inhibitors of phagolysosome acidification and the positive correlation between acidification and expression of IL-10. Further studies will be required to establish the mechanistic link between PDH, acidification and IL-10 expression.

Having pathways optimized for either aerobic or anaerobic growth reflects an ability to adapt to the diverse niches *S*. *pyogenes* encounters during infection at multiple tissue sites [31,50]. Cell death, the loss of perfusion, oxygen consumption by neutrophils, and the accumulation of inflammatory debris can create an anaerobic environment in suppurative sub-cutaneous lesions, which likely explains why the anaerobic (PFL), but not the aerobic (PDH), arm of mixed-acid fermentation is required for growth in the murine ulcer model. However, PDH plays an important role in regulating the growth/damage balance, making it an attractive target for therapeutic intervention in severe invasive infections. These diseases are notoriously difficult to treat due to extensive and rapid tissue destruction that limits perfusion of antibiotics to the site of bacterial multiplication, often necessitating multiple rounds of aggressive and disfiguring surgical interventions [51]. Thus, therapies that limit tissue destruction would reduce the need for surgeries and would improve pharmacodynamics of standard-of-care antibiotics to improve treatment outcomes. Inhibitors of PDH that improve treatment of bacterial infections have been reported [52] and additional prokaryotic-specific PDH inhibitors are under development [53]. This strategy may have a wider application, as recent studies have indicated that PDH, but not homo-lactic fermentation, is also important for *S*. *pyogenes* fitness in human blood, necrotizing myositis [54] and for streptococcal infection of swine [55]. Taken together, this work has shown how an analysis of bacterial metabolism can reveal a druggable bacterial target involved in disease tolerance that may act to limit tissue damage to improve treatment outcomes for severe tissue destructive disease.

## Materials and methods

### Ethics statement

All studies and procedures were approved by the Animal Studies Committee at Washington University School of Medicine (#22–0307, Animal Welfare Assurance #D16-00245) and were designed to limit the number of animals needed and minimize animal discomfort. The personnel performing the animal studies are expertly trained and approved to work with vertebrate animals.

### Bacterial strains

Culture of *E*. *coli* was in Luria-Bertani (LB) broth and *S*. *pyogenes* was cultured in Todd Hewitt + 1% Yeast Extract (THY) broth [56] or C medium supplemented with 0.2% glucose [56], where indicated. For *S*. *pyogenes*, shaken (aerated) growth conditions were generated using 5 ml of media in 18x150 mm borosilicate culture tubes with unsealed lids that were placed at a 45° angle in an orbital shaker rotating at 200x rpm with incubation at 37°C. Static (oxygen-limited) cultures were grown using 10 ml of media in 15 ml screw cap conical tubes with tightly sealed lids incubated while stationary at 37°C. Quantitation of *S*. *pyogenes* CFUs utilized THY medium solidified by the addition of 1.4% agar [56], which were incubated at 37°C while exposed to ambient air (aerobic conditions) or anaerobically using a commercial atmospheric container (GasPak EZ, catalog #BD 260001). Where indicated, media were buffered by the addition HEPES to a final concentration of 100 mM. All cultures were seeded from overnight cultures in C medium to an initial OD_600_ = 0.05, which were then incubated for the times indicated in the text. Where appropriate, culture media were supplemented with erythromycin at 1 μg/ml for *S*. *pyogenes* and 500 μg/ml for *E*. *coli*.

### Mutant construction

In-frame deletion mutations were constructed through homologous recombination at chromosomal loci using standard methods [57] and the temperature-sensitive shuttle vector pGCP213 [58]. Genomic loci are referenced according to the annotated genome of *S*. *pyogenes* HSC5 [27]. Plasmid constructs for in-frame deletion (**S4 Table**) were made by overlap PCR with the oligonucleotide primers (IDT, Coralville, IA) listed in **S5 Table**. Plasmid DNA was isolated using standard techniques and used to transform *S*. *pyogenes* as previously described [59]. Cis-complementation of *pdhA* was via insertion of the *pdhA* open-reading frame (see **Fig 1B**), including its predicted promoter into the chromosome of ΔPdh between the 5’ end of *guaB* and its predicted terminator, as described previously [60] and the primers listed in **S5 Table**. All constructs and deletions were validated through PCR and DNA sequencing (Genewiz, South Plainfield, NJ) using the appropriate primers (**S5 Table**).

### Analysis of growth

All cultures for measurement of growth were inoculated from cultures that were grown overnight in C medium under static conditions as described above. An exception was for experiments involving strain Only Pdh (**Fig 1**) which can only grow using the aerobic conditions described above. To initiate cultures for growth analysis, bacterial cells were harvested from overnight cultures by centrifugation, resuspended in 1/10 their original culture volume and seeded into fresh THY or C medium with or without 0.2% glucose to an initial OD_600_ = 0.05. Aerated and oxygen-limited growth conditions were generated as described above and growth measured by the increase in OD_600_ over time and by removing aliquots that were serially diluted and plated for determination of CFUs in parallel with plates incubated as described above. Where shown, representative images of plate cultures were taken following 24 hrs of culture.

### Murine subcutaneous ulcer model of infection

Infection of 6–8-week-old female SKH1-E, C57BL/6 and C57BL/6 IL-10^-/-^ (The Jackson Laboratory, B6.129P2-Il10^tm1Cgn^/J, strain#002251) mice was carried out following a well-established protocol [28]. Briefly, mice received a subcutaneous injection of ~10^7^ CFU of the indicated bacterial strains and the areas of the resulting ulcers determined following 72 hrs of infection from analysis of digital images using ImageJ as described [28]. Bacterial burdens in lesions were assessed by excision and homogenization of infected tissues and spot plating aliquots of the tissue homogenate as described [60]. Incubation was at 37^°^C under anaerobic conditions, with the exception that aerobic incubation was used for those strains not capable of anaerobic growth. All animal experiment were approved by the Institutional Animal Care and Use Committee (protocol #16–1119). Procedures were performed according to all institutional policies, Animal Welfare Act, NIH guidelines and American Veterinary Medical Association guidelines on euthanasia.

### Macrophage infection

RAW264.7 macrophages (ATCC, cat.#TIB-71) were grown in DMEM (Dulbecco’s Modified Eagles Medium (Sigma) with 10% fetal bovine serum) supplemented with antibiotics (1% Penicillin-Streptomycin) at 37^°^C in an atmosphere of 5% CO_2_. For infection, the bacterial inoculum was prepared as described above for murine infection and after replacing media with antibiotic free media, bacteria were added to macrophages cultured in 12-well dishes at an MOI of 10:1 which were then immediately subjected to centrifugation at 500 x g for 1 min. and placed in incubator for 1 hr. Wells were then washed twice with Dulbecco’s PBS, then DMEM containing antibiotics (100μg/ml Gentamicin) was added for 5 minutes and washed 3x with PBS before the cells incubated for an additional 2–4 hrs in plain DMEM. Cells were harvested using a cell scraper and collected by centrifugation at 1,000 x g for 5 min, were resuspended in 1ml water at pH 11 and lysed using a cup sonicator for 20 sec. Bacteria in the resulting suspension were collected by centrifugation (6,000 x g, 5 min) and resuspended in 100μl of C-media for determination of CFUs. Where indicated, cultures were treated with 0.1μM Bafilomycin A1, which is non-cytotoxic at this concentration [36]. Net fold-change in CFU was determined by comparison to cells harvested after the addition of antibiotic-containing medium. BMDM were infected by the same method and were prepared as described in **S3 Fig**. Data presented are pooled from 6 biological replicates conducted in 2 independent experiments.

### Macrophage trafficking and acidification

Bacteria were prepared as described above, labeled with Cell Tracker Green CMFDA as previously described [61] and then resuspended in 50 ml of fresh THY medium. Following an additional 30 min incubation at 37°C, bacteria were used to infect Raw264.7 cells as described above. At 5 min. prior to the 2 hr time point, cells were stained with LysoTracker Red DND-99 (Invitrogen, cat.#L7258) as previously described [62]. After an additional 5 min incubation, cells were washed twice with DPBS, fixed with 2% paraformaldehyde and mounted immediately in ProLong Diamond antifade (Invitrogen, cat.#36965) on glass slides and incubated 18 hours in the dark. Samples were then examined using a Leica model DM IRE 2 fluorescent microscope and images captured using a QImaging Retiga 1350 EX charged-coupled device camera and OpenLab software (Improvision, version 2.7). Quantitative analyses of co-localization were performed using ImageJ. Blinded images of infected macrophages were quantified for bacteria [G, green puncta] and bacteria + acidic vacuole [Y, yellow puncta] and co-localization determined by the following equation: [Y/(G+Y)] x 100 = % total bacteria co-localized with acidic vacuoles. Data presented for each condition represents the mean and standard error of the mean derived from at least 3 independent experiments and examination of a minimum of 1000-stained cells. Images were processed for publication using Adobe Photoshop (version 26.3.1).

### Transmission electron microscopy

Raw264.7 cell infected as described above were fixed in 4% paraformaldehyde/0.05% glutaraldehyde in 100mM PIPES/0.5mM MgCl_2_, pH 7.2 for 1 hr at 4°C, were embedded in 10% gelatin and then infiltrated overnight with 2.3M sucrose/20% polyvinyl pyrrolidone in PIPES/MgCl_2_ at 4°C. Samples were trimmed, frozen in liquid nitrogen, and sectioned with a Leica Ultracut FC7 cryo-ultramicrotome (Leica Microsystems Inc., Bannockburn, IL). Ultrathin sections of 65 nm were blocked with 5% FBS/5% NGS for 30 min and subsequently incubated with rat anti-LAMP1 antibody (Abcam, cat.#ab25245) for 1 hr at room temperature. Following washes in block buffer, sections were incubated with 18nm colloidal gold-conjugated goat anti-rat IgG (H+L) (Jackson Immuno-Research Labs, cat.#112-005-003) for 1 hr. Sections stained with 0.3% uranyl acetate/2% methyl cellulose and microscopy performed on a JEOL 1200 EX transmission electron microscope (JEOL USA Inc., Peabody, MA) equipped with an AMT 8 megapixel digital camera and AMT Image Capture Engine V602 software. All labeling experiments were conducted in parallel with controls omitting the primary antibody.

### Cytokine analyses

Supernatants were harvested from RAW264.7 cells following 4 hrs of infection and stored at -80^°^C. The following day, the concentration of IL-10 and TNFα were determined by ELISA according to the manufacturer’s protocol (R&D Systems, cat.#DY417, #DY410). Concentrations were determined by comparison to a standard curve generated using purified proteins.

### Measurement of SCFAs

Following 24 hrs of incubation in C-medium + 0.2% glucose, culture supernatants were prepared by centrifugation. Acetate, lactate and formate concentrations in supernatants were measured by a colorimetric assay using commercial kits following the manufacturer’s protocol (Sigma-Aldrich, cat.#MAK086, #MAK064, #MAK059).

### RNA-sequencing

Total RNA from Mock-, WT-, and ΔPdh- infected RAW264.7 cells (4 hrs post-infection) were isolated using Direct-Zol RNA MiniPrep Plus kit (Zymo Research, cat.#R2071) per the manufacture’s protocol. RNA samples were quantified using a Qubit 2.0 Fluorometer (Life Technologies, Carlsbad, CA, USA) and RNA integrity checked using an Agilent TapeStation 4200 (Agilent Technologies, Palo Alto, CA, USA). RNA sequencing libraries were prepared using the NEBNext Ultra II RNA Library Prep Kit for Illumina (cat.#E7760S) using manufacturer’s instructions. Briefly, mRNAs were first enriched with Oligo(dT) beads and then fragmented for 15 minutes at 94°C. First- and -second strand cDNAs were subsequently synthesized from cDNA fragments that were end-repaired, adenylated at 3’ends and ligated to universal adapters, followed by index addition and library enrichment by limited-cycle PCR. Libraries were validated on the Agilent TapeStation and quantitated using a Qubit 2.0 Fluorometer as above, and by quantitative PCR using a KAPA library quantification kit (Kapa Systems Roche, cat.#KK4824). Libraries were sequenced using an Illumina HiSeq 4000 using a 2x150bp Paired End (PE) configuration. Image analysis and base calling were conducted by the HiSeq Control Software. Raw sequence data (.bcl files) generated from Illumina HiSeq was converted into fastq files and de-multiplexed using bcl2fastq (Illumina, version2.17). One mismatch was allowed for index sequence identification.

### RNA-sequencing analysis

Sequence reads were trimmed using Trimmomatic [63] (version 0.36) to remove adapter sequences and nucleotide reads of poor quality, which were then mapped to the *Mus musculus* reference genome available on ENSEMBL using STAR aligner [64] (version 2.5.2b). Unique gene hit counts were generated from the resulting BAM files using the Counts feature of the Subread package [65] (version 1.5.2). Differential gene expression between groups was assessed using DESeq2 [66] (version 3.15) with the Wald test used to generate *P*-values and Log_2_ fold-changes. Genes with adjusted values for *P* < 0.05 and absolute log_2_ fold-changes > 1.0 were called as differentially expressed. List of genes significantly up- or down-regulated were subjected to pathway enrichment analysis using Reactome [67], with the probability that a specific pathway is over-represented calculated using a binomial test with *P*-values corrected for multiple testing using the Benjamini–Hochberg procedure, as described [67]. Raw data files for RNASeq are deposited in the NCBI BioSample database (http://www.ncbi.nlm.nih.gov/biosample/) under the ID number PRJNA904329.

### Statistical analyses

Unless otherwise indicated above, data presented from *in vitro* experiments represents the mean and standard error of the mean derived from at least three independent experiments, with differences between groups tested for significance by a two-tailed t-test. For the murine subcutaneous infection model, differences in lesion size and CFU counts between WT and mutant strains were derived from at least two independent experiments, each of which consisted of 5 mice per group, with differences tested for significance with the Mann-Whitney test. Test statistics were calculated with the InStat module of Prism (GraphPad, version 3.06). For all tests, the null hypothesis was rejected for *P*-values of >0.05, with the dimensions of specific comparisons indicated in the Figures, as defined in each Figure Legend.

## Supporting information

S1 Fig *S*. *pyogenes* Oxygen-consuming Central Carbon Metabolism. In the presence of oxygen, following the exhaustion of glucose, the lactate produced by homolactic fermentation (shown in blue) can be recovered to pyruvate (shown in red) by the oxygen-consuming enzyme Lactate Oxidase (LctO), which is further metabolized by Pyruvate Dehydrogenase (PDHC) to produce acetate and an additional molecule of ATP, as shown in red. Redox balance is maintained by the oxygen-consuming enzyme NADH Oxidase (Nox) and by the conversion of Acetyl-CoA to ethanol. Abbreviations for the other enzymes shown are as noted in Figure.
(PDF)

S2 Fig Complementation of ΔPdh. Growth of Wild type (**WT**), the PDH deletion mutant (**ΔPdh**) and ΔPdh complemented by insertion of the intact *pdhA* open-reading frame into the chromosomal *guaB* locus **(ΔPdh::*pdh***) under (**A**) oxygen-limited (Static) and (**B**) aerobic (Shaken) conditions, in the presence (+Glu) or absence (-Glu) of glucose supplementation (0.2%) is shown. Virulence of WT, mutant and complemented mutant strains were compared using subcutaneous infection of SKH1 mice with assessment of (**C**) ulcer lesion area (Damage) and (**D**) bacterial burden (Growth) in tissue at Day 3 post-infection. Growth of the strains in Raw264.7 macrophages was compared (**E**) along with the production of cytokines IL-10 (**F**) and TNFα (**G**) as determined by ELISA of supernatants from infected macrophages.
(PDF)

S3 Fig Infection of bone marrow-derived macrophages recapitulates phenotypes observed in Raw264.7 cells. Bone marrow-derived macrophages (BMDM) were generated from femurs of C57BL6/J mice by flushing femurs using PBS with a 27G needle. Cells were harvested by centrifugation (1,400 x rpm, 5 min, 4^°^C), resuspended in 5 ml of lysis buffer (155 mM NH_4_Cl) for 5 min. and then passed through 70 μm cell strainer. Cells were plated at a density of 4x10^6^ cells/ml in a 10-cm Petri dish and were differentiated by the addition of 20 ng/ml MCSF. A FACS analysis using F4/80 markers confirmed that >95% of the cells had differentiated into macrophages. These cells were then infected as described for Raw264.7 cells in the Materials and Methods. Assessed was the net change in bacterial viability (**A**), the production of IL-10 and TNFα (**B, C**), both determined by ELISA of supernatants from infected macrophages, and the Ratio of IL-10 vs TNFα (**D**). Where indicated, data presented represents the mean and standard error of the mean derived from at least 3 independent experiments. ***, P< 0.005; n.s., not significant.
(PDF)

S4 Fig Infected Raw264.7 cells are viable 4 hours post-infection. Raw264.7 macrophages were infected as described in the Materials and Methods. At the time when CFUs and cytokine expression was determined (4 hrs post-infection), the viability of WT- and ΔPdh-infected cells was determined by staining with a vital stain (Live/Dead^tm^, cat.# R37601, ThermoFisher Scientific) as directed by the manufacturer. Examination by fluorescent microscopy revealed that cells infected by either *S*. *pyogenes* strain were >95% viable (viable cells appear green, non-viable cells are red). Images are representative fields from a single experiment that was repeated 3 times.
(PDF)

S5 Fig PDH contributes to acid stress resistance. (**A**) The ability of the indicated strains (as described in the S2 Fig legend) to resist acid stress was determined by resuspending growing cultures in C media whose pH was adjusted as shown. The net change in viability (CFU/ml) was then determined following a 1 hr incubation. The growth yields of cultures under (**B**) aerobic (Shaken) or (**C**) oxygen-limited (Static) conditions were determined following overnight incubation. Where indicated by the (+), cultures were buffered to pH 7.4 using HEPES or were supplemented by Catalase. LOD, limit of detection. Where indicated, data presented represents the mean and standard error of the mean derived from at least 2 independent experiments. *, *P* < 0.05; **, *P* < 0.05; ****, *P* < 0.001.
(PDF)

S6 Fig Deletion of SLO does not phenocopy deletion of PDH. Cultured Raw264.7 cells were infected by WT, ΔPdh or a strain with a deletion in the gene encoding the secreted pore-forming cytolysin SLO (ΔSLO). Assessed was (**A**) the net change in bacterial viability, the production of (**B**) IL-10, (**C)** TNFα (both determined by ELISA of supernatants from infected macrophages) and (**D**) the Ratio of IL-10 vs TNFα. Where indicated, data presented represents the mean and standard error of the mean derived from at least 3 independent experiments. *, *P* < 0.05; ****, *P* < 0.001; n.s., not significant.
(PDF)

S1 Table Strains used in this study. (PDF)

S2 Table Genes in Raw267.4 cells differentially expressed between infection by ΔPdh and WT. (PDF)

S3 Table The 25 most relevant pathways identified by Reactome. (PDF)

S4 Table Plasmids used in this study. (PDF)

S5 Table Mutagenic, complementation and RT-PCR Primers used in this study. (PDF)

## References

[1] De Filippis F, Pasolli E, Ercolini D. The food-gut axis: lactic acid bacteria and their link to food, the gut microbiome and human health. FEMS Microbiol Rev. 2020;44(4):454–89. doi: 10.1093/femsre/fuaa015 32556166 PMC7391071

[2] George F, Daniel C, Thomas M, Singer E, Guilbaud A, Tessier FJ, et al. Occurrence and dynamism of lactic acid bacteria in distinct ecological niches: A multifaceted functional health perspective. Front Microbiol. 2018;9:2899. doi: 10.3389/fmicb.2018.02899 30538693 PMC6277688

[3] Martinussen J, Solem C, Holm AK, Jensen PR. Engineering strategies aimed at control of acidification rate of lactic acid bacteria. Curr Opin Biotechnol. 2013;24(2):124–9. doi: 10.1016/j.copbio.2012.11.009 23266099

[4] Neijssel OM, Snoep JL, Teixeira de Mattos MJ. Regulation of energy source metabolism in streptococci. J App Microbiol. 1997;83:12S–9S. 10.1046/j.1365-2672.83.s1.2.x 28621892

[5] Wang Y, Wu J, Lv M, Shao Z, Hungwe M, Wang J, et al. Metabolism characteristics of lactic acid bacteria and the expanding applications in food industry. Front Bioeng Biotechnol. 2021;9:612285. doi: 10.3389/fbioe.2021.612285 34055755 PMC8149962

[6] Zaunmüller T, Eichert M, Richter H, Unden G. Variations in the energy metabolism of biotechnologically relevant heterofermentative lactic acid bacteria during growth on sugars and organic acids. Appl Microbiol Biotechnol. 2006;72(3):421–9. doi: 10.1007/s00253-006-0514-3 16826375

[7] Aldunate M, Srbinovski D, Hearps AC, Latham CF, Ramsland PA, Gugasyan R, et al. Antimicrobial and immune modulatory effects of lactic acid and short chain fatty acids produced by vaginal microbiota associated with ubiosis and bacterial vaginosis. Front Physiol. 2015;6:164. 26082720 10.3389/fphys.2015.00164 PMC4451362

[8] van der Hee B, Wells JM. Microbial regulation of host physiology by short-chain fatty acids. Trends Microbiol. 2021;29(8):700–12. doi: 10.1016/j.tim.2021.02.001 33674141

[9] Carapetis JR, Steer AC, Mulholland EK, Weber M. The global burden of group A streptococcal diseases. Lancet Infect Dis. 2005;5(11):685–94. doi: 10.1016/S1473-3099(05)70267-X 16253886

[10] Sanyahumbi AS, Colquhoun S, Wyber R, Carapetis JR. Global Disease Burden of Group A Streptococci. In: Ferretti JJ, Stevens DL, Fischetti VA, editors. *Streptococcus pyogenes*: Basic Biology to Clinical Manifestations. Oklahoma City, OK: University of Oklahoma Health Sciences Center; 2016. 26866218

[11] Kreikemeyer B, McIver KS, Podbielski A. Virulence factor regulation and regulatory networks in *Streptococcus pyogenes* and their impact on pathogen-host interactions. Trends Microbiol. 2003;11(5):224–32. 12781526 10.1016/s0966-842x(03)00098-2

[12] Vega LA, Malke H, McIver KS. Virulence-related transcriptional regulators of *Streptococcus pyogenes*. In: Ferretti JJ, Stevens DL, Fischetti VA, editors. *Streptococcus pyogenes*: Basic Biology to Clinical Manifestations. Oklahoma City (OK): University of Oklahoma Health Sciences Center; 2016 26866215

[13] Cho KH, Caparon MG. Patterns of virulence gene expression differ between biofilm and tissue communities of *Streptococcus pyogenes*. Mol Microbiol. 2005;57(6):1545–56. 16135223 10.1111/j.1365-2958.2005.04786.x

[14] Graham MR, Virtaneva K, Porcella SF, Gardner DJ, Long RD, Welty DM, et al. Analysis of the transcriptome of group A Streptococcus in mouse soft tissue infection. Am J Pathol. 2006;169(3):927–42. doi: 10.2353/ajpath.2006.060112 16936267 PMC1698835

[15] Kietzman CC, Caparon MG. Distinct time-resolved roles for two catabolite-sensing pathways during *Streptococcus pyogenes* infection. Infect Immun. 2011;79:812–21. 21098101 10.1128/IAI.01026-10 PMC3028826

[16] Loughman JA, Caparon M. Regulation of SpeB in *Streptococcus pyogenes* by pH and NaCl: a model for *in vivo* gene expression. J Bacteriol. 2006;188(2):399–408. 16385029 10.1128/JB.188.2.399-408.2006 PMC1347310

[17] Pancholi V, Caparon M. *Streptococcus pyogenes* Metabolism. In: Ferretti JJ, Stevens DL, Fischetti VA, editors. *Streptococcus pyogenes*: Basic Biology to Clinical Manifestations. Oklahoma City (OK): University of Oklahoma Health Sciences Center; 2016. 26866220

[18] Diard M, Hardt WD. Evolution of bacterial virulence. FEMS Microbiol Rev. 2017;41(5):679–97. doi: 10.1093/femsre/fux023 28531298

[19] Kietzman CC, Caparon MG. CcpA and LacD.1 affect temporal regulation of *Streptococcus pyogenes* virulence genes. Infect Immun. 2010;78(1):241–52. 19841076 10.1128/IAI.00746-09 PMC2798178

[20] Kinkel TL, McIver KS. CcpA-mediated repression of streptolysin S expression and virulence in the group A streptococcus. Infect Immun. 2008;76(8):3451–63. doi: 10.1128/IAI.00343-08 18490461 PMC2493232

[21] Shelburne SA, Keith D 3rd, Horstmann N, Sumby P, Davenport MT, Graviss EA, et al. A direct link between carbohydrate utilization and virulence in the major human pathogen group A Streptococcus. Proc Natl Acad Sci USA. 2008;105(5):1698–703. doi: 10.1073/pnas.0711767105 18230719 PMC2234207

[22] Paluscio E, Watson ME Jr, Caparon MG. CcpA Coordinates growth/damage balance for *Streptococcus pyogenes* pathogenesis. Sci Rep. 2018;8(1):14254. 30250043 10.1038/s41598-018-32558-0 PMC6155242

[23] Deutscher J, Francke C, Postma PW. How phosphotransferase system-related protein phosphorylation regulates carbohydrate metabolism in bacteria. Microbiol Mol Biol Rev. 2006;70(4):939–1031. doi: 10.1128/MMBR.00024-06 17158705 PMC1698508

[24] Hutcherson JA, Sinclair KM, Belvin BR, Gui Q, Hoffman PS, Lewis JP. Amixicile, a novel strategy for targeting oral anaerobic pathogens. Sci Rep. 2017;7(1):10474. doi: 10.1038/s41598-017-09616-0 28874750 PMC5585216

[25] Snoep JL, van Bommel M, Lubbers F, Teixeira de Mattos MJ, Neijssel OM. The role of lipoic acid in product formation by *Enterococcus faecalis* NCTC 775 and reconstitution *in vivo* and *in vitro* of the pyruvate dehydrogenase complex. J Gen Microbiol. 1993;139 Pt 6:1325–9. 8360624 10.1099/00221287-139-6-1325

[26] Yamada T, Takahashi-Abbe S, Abbe K. Effects of oxygen on pyruvate formate-lyase *in situ* and sugar metabolism of *Streptococcus mutans* and *Streptococcus sanguis*. Infect Immun. 1985;47:129–34. 3965391 10.1128/iai.47.1.129-134.1985 PMC261487

[27] Port GC, Paluscio E, Caparon MG. Complete genome sequence of emm Type 14 *Streptococcus pyogenes* strain HSC5. Genome Announc. 2013;1(4):00612–13. 10.1128/genomeA.00612-13 PMC3744678 23950122

[28] Brenot A, King KY, Janowiak B, Griffith O, Caparon MG. Contribution of glutathione peroxidase to the virulence of *Streptococcus pyogenes*. Infect Immun. 2004;72(1):408–13. 14688122 10.1128/IAI.72.1.408-413.2004 PMC344014

[29] Bunce C, Wheeler L, Reed G, Musser J, Barg N. Murine model of cutaneous infection with gram-positive cocci. Infect Immun. 1992;60(7):2636–40. doi: 10.1128/iai.60.7.2636-2640.1992 1612733 PMC257214

[30] Gera K, McIver KS. Laboratory growth and maintenance of *Streptococcus pyogenes* (the Group A Streptococcus, GAS). Curr Protoc Microbiol. 2013;30:9d.2.1–9d.2.13. 10.1002/9780471729259.mc09d02s30 PMC3920295 24510893

[31] Watson MEJ, Neeley MN, Caparon MG. Animal models of *Streptococcus pyogenes* infection. In: Ferretti JJ, Stevens DL, Fischetti VA, editors. *Streptococcus pyogenes*: Basic biology to clinical Manifestations. Oklahoma City: Univ. Oklahoma Health Science Center; 2016.

[32] Mishalian I, Ordan M, Peled A, Maly A, Eichenbaum MB, Ravins M, et al. Recruited macrophages control dissemination of group A Streptococcus from infected soft tissues. J Immunol. 2011;187(11):6022–31. doi: 10.4049/jimmunol.1101385 22025550

[33] Bastiat-Sempe B, Love JF, Lomayesva N, Wessels MR. Streptolysin O and NAD-glycohydrolase prevent phagolysosome acidification and promote group A Streptococcus survival in macrophages. MBio. 2014;5(5):e01690–14. doi: 10.1128/mBio.01690-14 25227466 PMC4172074

[34] Lu SL, Kuo CF, Chen HW, Yang YS, Liu CC, Anderson R, et al. Insufficient acidification of autophagosomes facilitates Group A Streptococcus survival and growth in endothelial Cells. mBio. 2015;6(5):e01435–15. doi: 10.1128/mBio.01435-15 26419882 PMC4611045

[35] Sakurai A, Maruyama F, Funao J, Nozawa T, Aikawa C, Okahashi N, et al. Specific behavior of intracellular *Streptococcus pyogenes* that has undergone autophagic degradation is associated with bacterial streptolysin O and host small G proteins Rab5 and Rab7. J Biol Chem. 2010;285(29):22666–75. 20472552 10.1074/jbc.M109.100131 PMC2903418

[36] Conboy IM, Manoli D, Mhaiskar V, Jones PP. Calcineurin and vacuolar-type H+-ATPase modulate macrophage effector functions. Proc Natl Acad Sci USA. 1999;96(11):6324–9. doi: 10.1073/pnas.96.11.6324 10339586 PMC26880

[37] Gillespie M, Jassal B, Stephan R, Milacic M, Rothfels K, Senff-Ribeiro A, et al. The reactome pathway knowledgebase 2022. Nucleic Acids Research. 2021;50(D1):D687–D92. 10.1093/nar/gkab1028 PMC8689983 34788843

[38] Ouyang W O’Garra A. IL-10 Family cytokines IL-10 and IL-22: from basic science to clinical translation. Immunity. 2019;50(4):871–91. 30995504 10.1016/j.immuni.2019.03.020

[39] Hashem RM, Mahmoud MF, El-Moselhy MA, Soliman HM. Interleukin-10 to Tumor Necrosis Factor-alpha ratio is a predictive biomarker in nonalcoholic fatty liver disease: Interleukin-10 to Tumor Necrosis Factor-alpha ratio in steatohepatitis. Eur J Gastroenterol Hepatol. 2008;20(10):995–1001. doi: 10.1097/MEG.0b013e3282fdf65f 18787467

[40] Smith PM, Howitt MR, Panikov N, Michaud M, Gallini CA, Bohlooy-Y. M, et al. The microbial metabolites, short chain fatty acids, regulate colonic Treg homeostasis. Science. 2013;341:569–73. 23828891 10.1126/science.1241165 PMC3807819

[41] Timmer AM, Timmer JC, Pence MA, Hsu LC, Ghochani M, Frey TG, et al. Streptolysin O promotes group A Streptococcus immune evasion by accelerated macrophage apoptosis. J Biol Chem. 2009;284(2):862–71. doi: 10.1074/jbc.M804632200 19001420 PMC2613605

[42] Krzyszczyk P, Schloss R, Palmer A, Berthiaume F. The role of macrophages in acute and chronic wound healing and interventions to promote pro-wound healing phenotypes. Front Physiol. 2018;9:419. doi: 10.3389/fphys.2018.00419 29765329 PMC5938667

[43] Van Dyken SJ, Locksley RM. Interleukin-4- and Interleukin-13-mediated alternatively activated macrophages: roles in homeostasis and disease. Annu Rev Immunol. 2013;31:317–43. doi: 10.1146/annurev-immunol-032712-095906 23298208 PMC3606684

[44] Schulthess J, Pandey S, Capitani M, Rue-Albrecht KC, Arnold I, Franchini F, et al. The short chain fatty acid butyrate imprints an antimicrobial program in macrophages. Immunity. 2019;50(2):432–45.e7. doi: 10.1016/j.immuni.2018.12.018 30683619 PMC6382411

[45] Soares MP, Gozzelino R, Weis S. Tissue damage control in disease tolerance. Trends Immunol. 2014;35(10):483–94. doi: 10.1016/j.it.2014.08.001 25182198

[46] Soares MP, Teixeira L, Moita LF. Disease tolerance and immunity in host protection against infection. Nat Rev Immunol. 2017;17(2):83–96. doi: 10.1038/nri.2016.136 28044057

[47] Mozola CC, Caparon MG. Dual modes of membrane binding direct pore formation by Streptolysin O. Mol Microbiol. 2015;97:1036–50. doi: 10.1111/mmi.13085 26059530 PMC4692278

[48] Mozola CC, Magassa N, Caparon MG. A novel cholesterol-insensitive mode of membrane binding promotes cytolysin-mediated translocation by Streptolysin O. Mol Microbiol. 2014;94:675–87. doi: 10.1111/mmi.12786 25196983 PMC4213296

[49] Korithoski B, Lévesque CM, Cvitkovitch DG. The involvement of the pyruvate dehydrogenase E1alpha subunit, in *Streptococcus mutans* acid tolerance. FEMS Microbiol Lett. 2008;289(1):13–9. 19054088 10.1111/j.1574-6968.2008.01351.x

[50] Efstratiou A, Lamagni T. Epidemiology of *Streptococcus pyogenes*. In: Ferretti JJ, Stevens DL, Fischetti VA, editors. *Streptococcus pyogenes*: Basic Biology to Clinical Manifestations. Oklahoma City (OK): University of Oklahoma Health Sciences Center; 2016. 26866208

[51] Misiakos EP, Bagias G, Patapis P, Sotiropoulos D, Kanavidis P, Machairas A. Current concepts in the management of necrotizing fasciitis. Front Surg. 2014;1:36. doi: 10.3389/fsurg.2014.00036 25593960 PMC4286984

[52] Birkenstock T, Liebeke M, Winstel V, Krismer B, Gekeler C, Niemiec MJ, et al. Exometabolome analysis identifies pyruvate dehydrogenase as a target for the antibiotic triphenylbismuthdichloride in multiresistant bacterial pathogens. J Biol Chem. 2012;287(4):2887–95. doi: 10.1074/jbc.M111.288894 22144679 PMC3268445

[53] Zhou Y, Zhang S, He H, Jiang W, Hou L, Xie D, et al. Design and synthesis of highly selective pyruvate dehydrogenase complex E1 inhibitors as bactericides. Bioorg Med Chem. 2018;26(1):84–95. doi: 10.1016/j.bmc.2017.11.021 29170025

[54] Le Breton Y, Belew AT, Valdes KM, Islam E, Curry P, Tettelin H, et al. Essential genes in the core genome of the human pathogen *Streptococcus pyogenes*. Sci Rep. 2015;5:9838. 25996237 10.1038/srep09838 PMC4440532

[55] Wang Y, Wang Y, Liu B, Wang S, Li J, Gong S, et al. pdh modulate virulence through reducing stress tolerance and biofilm formation of *Streptococcus suis* serotype 2. Virulence. 2019;10(1):588–99. 31232165 10.1080/21505594.2019.1631661 PMC6592368

[56] Port GC, Vega LA, Nylander AB, Caparon MG. *Streptococcus pyogenes* polymyxin B-resistant mutants display enhanced ExPortal integrety. J Bacteriol. 2014;196:2563–77. 24794568 10.1128/JB.01596-14 PMC4097577

[57] Cho KH, Port GC, Caparon M. Genetics of Group A Streptococci. Microbiol Spectrum 2019;7(2):7.2.01. doi: 10.1128/microbiolspec.GPP3-0056-2018 30825299 PMC11590684

[58] Nielsen HV, Guiton PS, Kline KA, Port GC, Pinkner JS, Neiers F, et al. The metal ion-dependent adhesion site motif of the *Enterococcus faecalis* EbpA pilin mediates pilus function in catheter-associated urinary tract infection. MBio. 2012;3(4):e00177–12. 22829678 10.1128/mBio.00177-12 PMC3419518

[59] Le Breton Y, McIver KS. Genetic manipulation of *Streptococcus pyogenes* (the Group A Streptococcus, GAS). Curr Protoc Microbiol. 2013;30:9d.3.1–9d.3.29. 10.1002/9780471729259.mc09d03s30 PMC3920291 24510894

[60] Port GC, Cusumano ZT, Tumminello PR, Caparon MG. SpxA1 and SpxA2 act coordinately to fine-tune stress responses and virulence in *Streptococcus pyogenes*. MBio. 2017;8(2): e00288–17. 28351920 10.1128/mBio.00288-17 PMC5371413

[61] Lin A, Loughman JA, Zinselmeyer BH, Miller MJ, Caparon MG. Streptolysin S inhibits neutrophil recruitment during the early stages of *Streptococcus pyogenes* infection. Infect Immun. 2009;77(11):5190–201. 19687200 10.1128/IAI.00420-09 PMC2772533

[62] Guerra F, Paiano A, Migoni D, Girolimetti G, Perrone AM, De Iaco P, et al. Modulation of RAB7A protein expression determines resistance to Cisplatin through late endocytic pathway impairment and extracellular vesicular secretion. Cancers (Basel). 2019;11(1):52. doi: 10.3390/cancers11010052 30626032 PMC6357196

[63] Bolger AM, Lohse M, Usadel B. Trimmomatic: a flexible trimmer for Illumina sequence data. Bioinformatics. 2014;30(15):2114–20. doi: 10.1093/bioinformatics/btu170 24695404 PMC4103590

[64] Dobin A, Davis CA, Schlesinger F, Drenkow J, Zaleski C, Jha S, et al. STAR: ultrafast universal RNA-seq aligner. Bioinformatics. 2013;29(1):15–21. doi: 10.1093/bioinformatics/bts635 23104886 PMC3530905

[65] Liao Y, Smyth GK, Shi W. The R package Rsubread is easier, faster, cheaper and better for alignment and quantification of RNA sequencing reads. Nucleic Acids Res. 2019;47(8):e47. doi: 10.1093/nar/gkz114 30783653 PMC6486549

[66] Love MI, Huber W, Anders S. Moderated estimation of fold change and dispersion for RNA-seq data with DESeq2. Genome Biol. 2014;15(12):550. doi: 10.1186/s13059-014-0550-8 25516281 PMC4302049

[67] Fabregat A, Sidiropoulos K, Viteri G, Forner O, Marin-Garcia P, Arnau V, et al. Reactome pathway analysis: a high-performance in-memory approach. BMC Bioinformatics. 2017;18(1):142. doi: 10.1186/s12859-017-1559-2 28249561 PMC5333408

