# Supplementary figures and images for: Central carbon flux controls growth/damage balance for Streptococcus pyogenes
Source: PLoS Pathog. 2023 Jun 29;19(6):e1011481. doi: 10.1371/journal.ppat.1011481 (PMC10337930; doi:10.1371/journal.ppat.1011481)

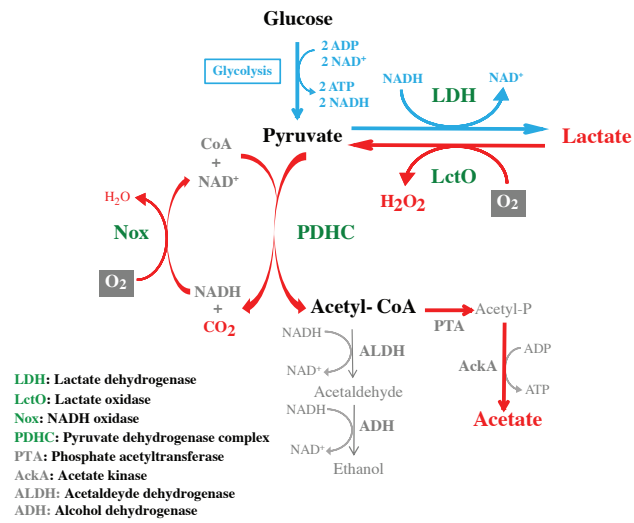

S1 Fig

Supplement: S1 Fig — In the presence of oxygen, following the exhaustion of glucose, the lactate produced by homolactic fermentation (shown in blue) can be recovered to pyruvate (shown in red) by the oxygen-consuming enzyme Lactate Oxidase (LctO), which is further metabolized by Pyruvate Dehydrogenase (PDHC) to produce acetate and an additional molecule of ATP, as shown in red. Redox balance is maintained by the oxygen-consuming enzyme NADH Oxidase (Nox) and by the conversion of Acetyl-CoA to ethanol. Abbreviations for the other enzymes shown are as noted in Figure. (PDF) [file ppat.1011481.s001.pdf]

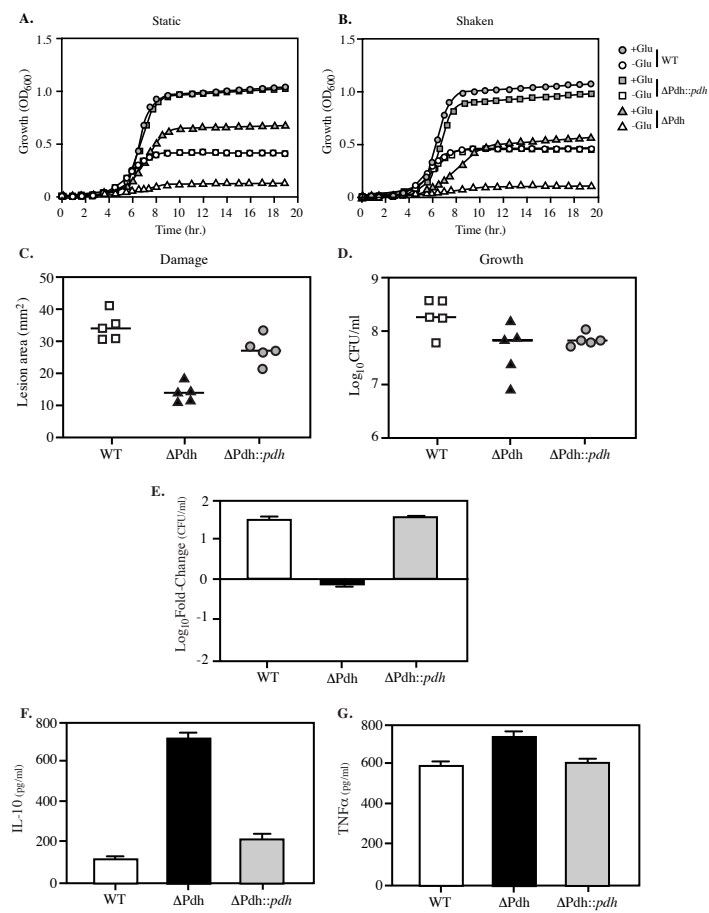

S2 Fig

Supplement: S2 Fig — Growth of Wild type (WT), the PDH deletion mutant (ΔPdh) and ΔPdh complemented by insertion of the intact pdhA open-reading frame into the chromosomal guaB locus (ΔPdh::pdh) under (A) oxygen-limited (Static) and (B) aerobic (Shaken) conditions, in the presence (+Glu) or absence (-Glu) of glucose supplementation (0.2%) is shown. Virulence of WT, mutant and complemented mutant strains were compared using subcutaneous infection of SKH1 mice with assessment of (C) ulcer lesion area (Damage) and (D) bacterial burden (Growth) in tissue at Day 3 post-infection. Growth of the strains in Raw264.7 macrophages was compared (E) along with the production of cytokines IL-10 (F) and TNFα (G) as determined by ELISA of supernatants from infected macrophages. (PDF) [file ppat.1011481.s002.pdf]

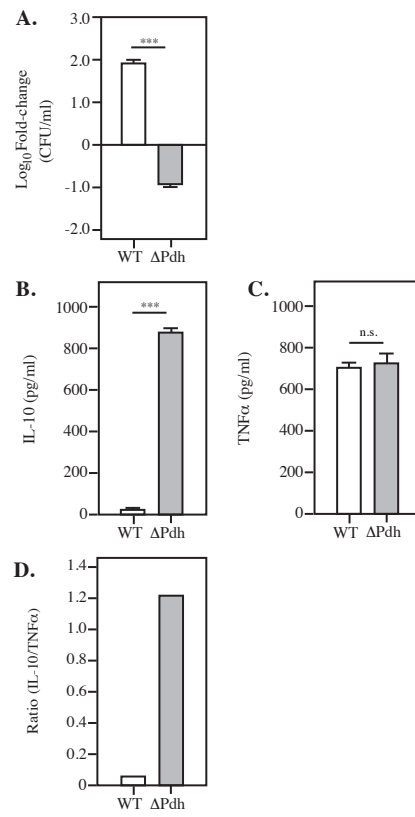

S3 Fig

Supplement: S3 Fig — Bone marrow-derived macrophages (BMDM) were generated from femurs of C57BL6/J mice by flushing femurs using PBS with a 27G needle. Cells were harvested by centrifugation (1,400 x rpm, 5 min, 4°C), resuspended in 5 ml of lysis buffer (155 mM NH4Cl) for 5 min. and then passed through 70 μm cell strainer. Cells were plated at a density of 4x10^6^ cells/ml in a 10-cm Petri dish and were differentiated by the addition of 20 ng/ml MCSF. A FACS analysis using F4/80 markers confirmed that >95% of the cells had differentiated into macrophages. These cells were then infected as described for Raw264.7 cells in the Materials and Methods. Assessed was the net change in bacterial viability (A), the production of IL-10 and TNFα (B, C), both determined by ELISA of supernatants from infected macrophages, and the Ratio of IL-10 vs TNFα (D). Where indicated, data presented represents the mean and standard error of the mean derived from at least 3 independent experiments. ***, P< 0.005; n.s., not significant. (PDF) [file ppat.1011481.s003.pdf]

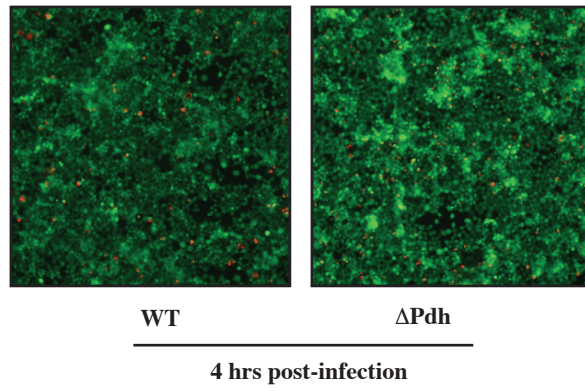

S4 Fig

Supplement: S4 Fig — Raw264.7 macrophages were infected as described in the Materials and Methods. At the time when CFUs and cytokine expression was determined (4 hrs post-infection), the viability of WT- and ΔPdh-infected cells was determined by staining with a vital stain (Live/Deadtm, cat.# R37601, ThermoFisher Scientific) as directed by the manufacturer. Examination by fluorescent microscopy revealed that cells infected by either S. pyogenes strain were >95% viable (viable cells appear green, non-viable cells are red). Images are representative fields from a single experiment that was repeated 3 times. (PDF) [file ppat.1011481.s004.pdf]

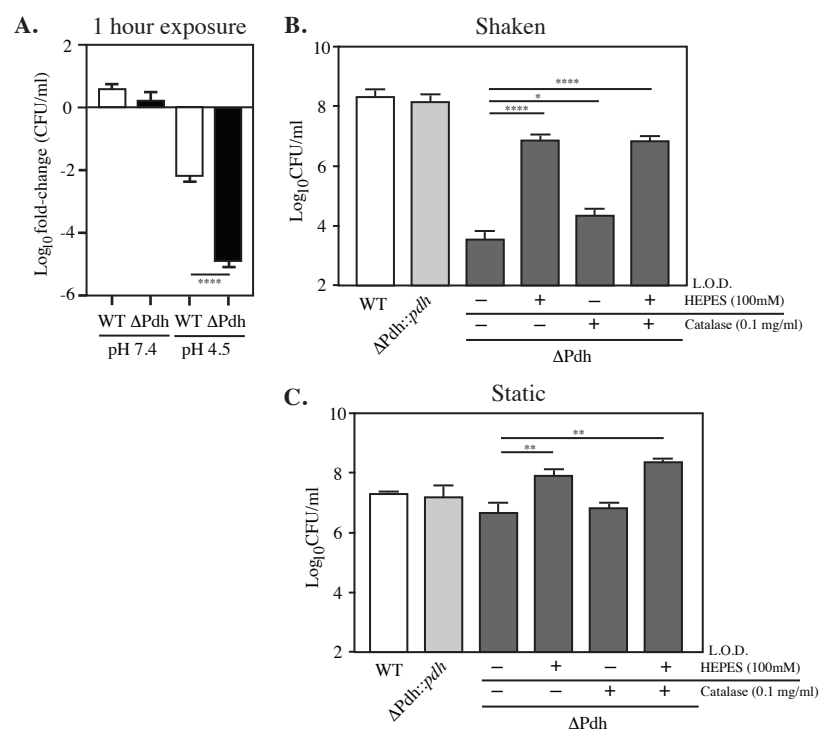

S5 Fig

Supplement: S5 Fig — (A) The ability of the indicated strains (as described in the S2 Fig legend) to resist acid stress was determined by resuspending growing cultures in C media whose pH was adjusted as shown. The net change in viability (CFU/ml) was then determined following a 1 hr incubation. The growth yields of cultures under (B) aerobic (Shaken) or (C) oxygen-limited (Static) conditions were determined following overnight incubation. Where indicated by the (+), cultures were buffered to pH 7.4 using HEPES or were supplemented by Catalase. LOD, limit of detection. Where indicated, data presented represents the mean and standard error of the mean derived from at least 2 independent experiments. *, P < 0.05; **, P < 0.05; ****, P < 0.001. (PDF) [file ppat.1011481.s005.pdf]

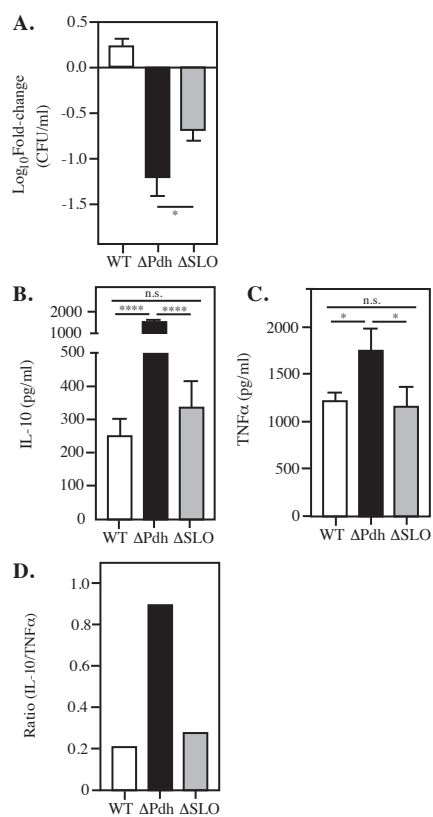

S6 Fig

Supplement: S6 Fig — Cultured Raw264.7 cells were infected by WT, ΔPdh or a strain with a deletion in the gene encoding the secreted pore-forming cytolysin SLO (ΔSLO). Assessed was (A) the net change in bacterial viability, the production of (B) IL-10, (C) TNFα (both determined by ELISA of supernatants from infected macrophages) and (D) the Ratio of IL-10 vs TNFα. Where indicated, data presented represents the mean and standard error of the mean derived from at least 3 independent experiments. *, P < 0.05; ****, P < 0.001; n.s., not significant. (PDF) [file ppat.1011481.s006.pdf]
