# Supplementary material for: Central carbon flux controls growth/damage balance for Streptococcus pyogenes
Source: PLoS Pathog. 2023 Jun 29;19(6):e1011481. doi: 10.1371/journal.ppat.1011481 (PMC10337930; doi:10.1371/journal.ppat.1011481)
Supplement: S1 Table — (PDF) [file ppat.1011481.s007.pdf]

Table S1. Strains used in this study

Strain (Alternative name)[1]	Relevant Genotype	Gene(s) Mutated[2]	Parental Strain	Mutagenic Plasmid[3]	Reference
HSC5 (WT)	Wild Type	-----	-----	-----	1
JAM037 (ΔPdh)	Δ*pdhA*	03920	HSC5	pJAM199	This study
JAM055 (ΔPfl)	Δ*pfl*	07865	HSC5	pEP101	This study
JAM129 (ΔLdh)	Δ*ldh*	04360	HSC5	pEP98	This study
JAM008 (Only Pfl)	Δ*ldh*Δ*pdhA*	04360/03920	JAM129	pJAM199	This study
JAM053 (Only Pdh)	Δ*ldh*Δ*pfl*	04360/07865	JAM129	pEP101	This study
JAM063 (Only Ldh)	Δ*pfl*Δ*pdhA*	07865/03920	JAM055	pJAM199	This study
JAM149 (ΔSlo)	Δ*slo*	00955	HSC5	pJAM147	This study
JAM093 (ΔPdh::Pdh)	Δ*pdhA*::*pdhA*	n/a	Δ*pdhA*	pJAM86	This study

[1]Strains referred to by alternative name in text.

[2]L897_xxxxx in the HSC5 genome (1), where xxxxx is the locus indicated in the Table.

[3]Mutagentic plasmid introduced into the indicated parental strain. See Materials and Methods.

REFERENCE

1. Port GC, Paluscio E, Caparon MG. 2013. Genome Announc. E00612-00613.
