# Supplementary material for: Central carbon flux controls growth/damage balance for Streptococcus pyogenes
Source: PLoS Pathog. 2023 Jun 29;19(6):e1011481. doi: 10.1371/journal.ppat.1011481 (PMC10337930; doi:10.1371/journal.ppat.1011481)
Supplement: S2 Table — (PDF) [file ppat.1011481.s008.pdf]

Table S2. Genes in Raw267.4 cells differentially expressed between infection by ΔPdh and WT

Gene ID	Log_2 Fold-Change	P adj	Gene name	Description/Function
00000000325	1.8931	9.55E-07	Arvcf	Involved in protein-protein interactions at adherens junctions.
00000000386	1.0129	4.01E-16	Mx1	Correlated with FOXP3 and IL-10.
00000000392	1.4523	6.30E-06	Fap	Knockdown of FAP in CAF9 cells attenuated migration of TE-9 cells and macrophage-like cells and suppressed expression of CD163 and IL10.
00000001131	1.5862	2.14E-05	Timp1	A novel IL-10 signalling mechanism regulates TIMP-1 expression, wound healing and tissue repair.
00000003541	1.0145	6.48E-05	Ier3	May play a role in the ERK signaling pathway
00000003545	3.6876	1.12E-05	Fosb	Suppress systemic inflammatory response to endotoxin, depedent on CREB
00000006403	1.0274	1.70E-02	Adamts4	ADAMTS-4 increased the production of IL-10
00000006445	1.6952	3.82E-14	Epha2	EphA2 expression may regulate the progression to advanced atherosclerosis by regulating smooth muscle proliferation and extracellular matrix deposition
00000011008	1.1925	7.03E-09	Mcoln2	MCOLN2 belongs to the transient receptor potential (TRP) protein superfamily, which consists of gated, tetrameric cation channels with diverse physiological functions, particularly in sensory signaling.
00000014599	1.1464	2.28E-20	Csf1	In tumors, expression of CSF1 was correlated with M2-like macrophages. CSF-1 has therapeutic potential in tissue repair.
00000015396	1.0106	3.50E-04	Cd83	A specific marker for mature dendritic cells
00000016283	1.0528	1.43E-03	H2-M2	MHC class Ib antigen
00000016529	1.5056	9.07E-04	Il10	Human cytokine synthesis inhibitory factor (CSIF), is an anti-inflammatory cytokine
00000018925	1.6923	1.71E-03	Heatr9	Heatr9 is induced by influenza virus and RSV. Heatr9 knockdown during viral infection was shown to affect chemokine expression.
00000018930	1.1305	9.15E-04	Ccl4	Resveratrol improves CCL4-induced liver fibrosis in mouse by upregulating endogenous IL-10 to reprogramme macrophages phenotype from M(LPS) to M(IL -4).
00000020108	1.4567	5.57E-09	Ddit4	IL-10 suppresses mammalian target of rapamycin (mTOR) activity through th induction of an mTOR inhibitor, DDIT4. IL-10 inhibits mTORC1 signalling in a STAT3–DDIT4- dependent manner
00000020423	1.1469	7.33E-27	Btg2	Enhanced BTG2 induces G2/M cell cycle arrest and apoptotic
00000020826	1.3178	3.48E-28	Nos2	IL-10 and NOS2 modulate antigen-specific reactivity and nerve infiltration by T cells in experimental leprosy. Expressions of IL10 and NOS2 (iNOS) were negatively correlated (P<0.001).
00000021367	1.7272	9.44E-04	Edn1	Endothelin-1 (ET-1), which is up-regulated during tissue repair and fibrosis, induces lung fibroblasts to produce and contract extracellular matrix
00000024486	1.8345	7.99E-06	Hbegf	HB-EGF levels increase in response to different forms of injuries as well as stimuli, such as lysophosphatidic acid, retinoic acid
00000025491	2.0027	7.64E-09	Ifitm1	Ifitm1 (Interferon induced transmembrane protein 1)
00000025746	1.7658	1.41E-04	Il6	Functions in inflammation and the maturation of B cells.
00000026817	1.2800	7.22E-13	Ak1	Plays an important role in cellular energy homeostasis and in adenine nucleotide metabolism
00000026981	1.1022	7.33E-37	Il1rn	Interleukin 1 Receptor Antagonist, This protein inhibits the activities of interleukin 1, alpha (IL1A) and interleukin 1, beta (IL1B)
00000027219	1.1501	4.39E-04	Slc28a2	Uptake and salvage of purine nucleosides in kidney and other tissues
00000027398	1.8828	1.12E-04	Il1b	Potent proinflammatory cytokine
00000027399	2.0150	7.82E-05	Il1a	Cell-to-cell communication
00000028211	1.9485	4.40E-22	Trp53inp1	Regulates cell cycle progression and apoptosis, antioxidant
00000028270	1.0913	6.21E-04	Gbp2	Protective immunity against microorganisms, Inhibition of Stat3 pathway prevents GBP2-promoted FN1 induction and cell invasion.
00000028341	1.4138	2.10E-09	Nr4a3	NR4A3 expression is also increased at sites of neutrophilic inflammation
00000028602	1.4454	6.64E-10	Tnfrsf8	CD30L (=CD153): T and B cell activation, TNF receptor superfamily
00000028716	1.2066	1.24E-05	Pdzk1ip1	Cargo protein that transports membrane proteins from the endoplasmic reticulum. triggering local inflammation
00000028967	1.2466	4.37E-25	Errfi1	mig6/errfi-1, a negative regulator of the EGFR family
00000030748	1.1722	4.05E-09	Il4ra	A deletion of IL-4Rα completely abrogates TB tissue pathology in these mice
00000031444	1.2707	1.89E-21	F10	Vitamin K-dependent coagulation factor X of the blood coagulation cascade
00000031779	1.0309	2.65E-04	Ccl22	High CCL22 controls had significantly elevated levels of anti-inflammatory Il 10compared with low CCL22 controls.
00000032578	2.3870	1.21E-12	Cish	Cytokine-induced STAT inhibitor (CIS), also known as suppressor of cytokine signaling (SOCS), or STAT-induced STAT inhibitor (SSI), protein family.
00000032584	1.8159	3.82E-06	Mst1r	Macrophage Stimulating 1 Receptor

00000034394	1.4041	2.02E-05	Lif	LIF confers protection against endotoxemia induced by the bacterial endotoxi lipopolysaccharide (LPS) by enhancing interleukin 10 (IL10) and inhibiting tumor necrosis factor alpha (TNFα) synthesis and release by Mφs
00000034457	1.7854	1.16E-15	Eda2r	Ectodysplasin A2 Receptor, EDA: control of hair follicle, sweat gland, teeth development, TNF receptor superfamily
00000035042	1.1900	2.31E-02	Ccl5	CCL5-induced the elevation of IL-10 expression, on Ang II-induced 12-lipoxygenase (LO) and endothelin (ET)-1 expression
00000035356	1.1324	2.95E-03	Nfkbiz	NF-kappa-B inhibitor zeta, Studies in mouse indicate that this gene product is one of the nuclear I kappa B proteins and an activator of IL-6 production.
00000035373	1.1654	1.95E-07	Ccl7	A secreted chemokine which attracts macrophages during inflammation and metastasis
00000037447	1.3123	2.93E-06	Arid5a	AT-Rich Interaction Domain 5A, As RNA-binding protein involved in the regulation of inflammatory response by stabilizing selective inflammation-related mRNAs, such as IL6, STAT3 and TBX21. Arid5a controls IL-6 mRNA stability, which contributes to elevation of IL-6 level in vivo
00000038067	1.3985	1.63E-02	Csf3	Colony stimulating factor 3 (CSF3)
00000038179	1.2124	2.05E-03	Slamf7	CS1 (also known as CD319, CRACC and SLAMF7) was identified as an NK cell receptor regulating immune functions.
00000040026	1.5036	4.96E-04	Saa3	The presence of SAA3 in the inflamed colon mucosal serves to protect epithelial barrier in part through expansion of IL-22-producing neutrophils.
00000040264	1.1319	6.14E-03	Gbp2b	Infection caused a large increase of Gbp2b/Gbp1 and Gbp5 expression
00000040663	1.2317	3.49E-06	Clcf1	In mice, CLCF1 induces B-cell expansion, enhances humoral responses and triggers autoimmunity. CLCF1 is another member of the IL-6-type cytokine
00000041801	1.9090	1.99E-29	Phlda3	Tumor suppressor.
00000043421	1.0397	1.96E-20	Hilpda	Hypoxia-inducible Lipid Droplet-associated (HILPDA) Is
00000043953	1.1893	1.38E-04	Ccrl2	Plays a critical role for the development of Th2 responses
00000044313	1.2662	2.70E-03	Mab21l3	Mab-21 Like 3
00000044701	1.2467	2.71E-02	Il27	Pleiotropic cytokine capable of influencing both innate and adaptive immune responses. With anti- and pro-inflammatory activity
00000044786	1.0368	2.39E-04	Zfp36	Strikingly, loss of ZFP36 in vivo accelerated T cell responses to acute viral infection and enhanced anti-viral immunity.
00000046031	1.2534	7.51E-03	Fam26f	FAM26F (family with sequence similarity 26, member F) is a recently identified tetraspanin-like membrane glycoprotein which is predicted to make homophilic interactions and potential synapses between several immune cells
00000046733	1.1894	4.99E-07	Gprc5a	Tumor suppressor
00000048458	1.7762	4.28E-11	Fam212b	Induced In Neural Crest By AP2-Alpha Protein-Related Homolog
00000051379	1.4969	1.92E-36	Flrt3	Flrt3 is induced by bacterial infection, implicated in neurite outgrowth and cel adhesion
00000053113	1.2719	1.66E-03	Socs3	STAT-induced STAT inhibitor (SSI)
00000053318	1.3644	7.44E-03	Slamf8	Anti-inflammatory, Slamf8 is a negative regulator of Nox2 activity in macrophages
00000053846	2.0451	2.71E-03	Lipg	Endothelial lipase (LIPG), LIPG is naturally acknowledged to be a biological factor to the lipid metabolism and atherosclerosis
00000054203	1.1756	5.91E-08	Ifi205	Regulates immune signaling via transcriptional regulation of the inflammason adapter ASC
00000054855	1.1527	7.84E-09	Rnd1	Rnd1 expression was found to be induced during inflammation in endothelial cells
00000058427	1.2694	1.60E-05	Cxcl2	Macrophage chemokines CXCL1/CXCL2 control the early stage of neutrophi recruitment during tissue inflammation.
00000060183	1.3732	4.66E-05	Cxcl11	Predominantly induced by interferon (IFN)-γ and share an exclusive chemokine receptor named CXC chemokine receptor 3 (CXCR3).
00000061878	1.0708	7.79E-08	Sphk1	Glucocorticoidss attenuate inflammation in a murine model of ALI by synergistic upregulation of SphK1 gene expression in macrophages; The induction of both LIGHT and SPHK1 mRNA was dependent on IL-10
00000062345	1.4446	1.08E-03	Serpinb2	Protects macrophages from apoptosis; Macrophage expression of serpinB2 is upregulated by LPS through a mechanism involving CREB and NFκB and is important for the maintenance of TLR4 activation, thereby preventing rapid macrophage death and premature cessation of the innate immune response; Recent studies have implicated an anti-inflammatory role for serpinB2 and it i considered to be part of the M2-associated gene
00000069662	1.8878	4.22E-07	Marcks	MARCKS constitutes 90% of all proteins synthesized in neutrophils in response to exposure to TNF-α or LPS; During the inflammatory response, MARCKS has also been shown to act as a major regulator of human neutrophil migration and adhesion
00000072844	1.2771	5.42E-04	5530011O06Ri	Lysosomal, immune-related, and lipid metabolic genes
00000078763	1.0555	4.45E-03	Slfn1	SLFN11 specifically blocks the production of retroviruses such as HIV-1
00000079138	1.0495	5.24E-03	Gm8818	

00000081219	1.2494	2.77E-03	Bambi-ps1	BMP and activin membrane-bound inhibitor, pseudogene
00000085178	1.0772	2.07E-03	Kdm6bos	KDM1 lysine (K)-specific demethylase 6B
00000087263	1.1072	2.86E-02	Gm15726	
00000090231	1.0374	1.37E-06	Cfb	Complement factor B
00000105504	1.3606	9.41E-07	Gbp5	Overexpression of GBP5 inhibited virus replication by enhancing the expression of virus-induced interferon (IFN) and IFN-related effectors
00000108436	1.0419	1.23E-02	Gm44851	

[1]Ensembl Mouse (GRCm39). https://useast.ensembl.org/Mus_musculus.

[2]Log_2-fold change of normalized mean hit counts derived from two independent experiments: $Log_2(\Delta Pdh/WT)$.

[3]Wald test p value.

[4]Benjamini-Hochberg false discovery rate adjusted p-value.
