# Supplementary material for: Central carbon flux controls growth/damage balance for Streptococcus pyogenes
Source: PLoS Pathog. 2023 Jun 29;19(6):e1011481. doi: 10.1371/journal.ppat.1011481 (PMC10337930; doi:10.1371/journal.ppat.1011481)
Supplement: S3 Table — (PDF) [file ppat.1011481.s009.pdf]

Table S3. The 25 most relevant pathways identified by Reactome[1].

Pathway Name	Entities[2]			Reaction		
	found	ratio	p-value	FDR[3]	found	ratio
Interleukin-10 signaling	27 / 86	0.006	1.11E-16	1.14E-14	12 / 15	0.001
Signaling by Interleukins	41 / 67	0.044	1.11E-16	1.14E-14	95 / 493	0.037
Cytokine Signaling in Immune system	51 / 1,108	0.075	1.11E-16	1.14E-14	109 / 687	0.052
Interleukin-4 and Interleukin-13 signaling	20 / 216	0.015	1.55E-15	1.20E-13	22 / 47	0.004
Immune System	52 / 2,713	0.184	4.00E-13	2.48E-11	139 / 1,593	0.012
Chemokine receptors bind chemokines	10 / 57	0.004	5.97E-11	3.04E-09	7 / 19	0.001
Peptide ligand-binding receptors	11 / 211	0.014	1.45E-06	6.39E-09	10 / 76	0.006
CLEC7A/inflammasome pathway	3 / 8	5.43E-05	4.30E-05	0.002	2 / 4	3.02E-04
Interleukin-6 family signaling	4 / 31	0.002	1.33E-04	0.005	22 / 34	0.003
Inhibition of nitric oxide production	2 / 5	3.40E-04	8.03E-04	0.025	2 / 5	3.78E-04
Interferon alpha/beta signaling	7 / 91	0.013	0.001	0.029	2 / 5	0.002
Interleukin-12 family signaling	5 / 96	0.007	0.001	0.03	11 / 114	0.009
PTK6 Activates STAT3	2 / 7	4.76E-04	0.002	0.036	3 / 9	6.80E-04
Class A/1 (Rhodopsin-like receptor	11 / 475	0.032	0.002	0.04	10 / 158	0.012
Interleukin-1 processing	2 / 8	5.43E-04	0.002	0.04	2 / 5	3.78E-04
Transport of gamma-carboxylated protein precursors from the endoplasmic reticulum to the Golgi apparatus	2 / 9	6.11E-04	0.003	0.048	2 / 9	6.80E-04
Removal of aminoterminal propeptides from gamma- carboxylated proteins	2 / 10	6.79E-04	0.003	0.051	2 / 9	6.80E-04

Gene and protein expression by JAK STAT signaling after Interleukin stimulation	4 / 73	0.005	0.003	0.051	2 / 36	0.003
CLEC7A (Dectin-1) signaling	5 / 120	0.008	0.003	0.051	3 / 45	0.003
RUNX1 regulates transcription of genes involved in differentiation of keratinocytes	2 / 11	7.47E-04	0.004	0.056	2 / 8	6.04E-04
Interleukin-12 signaling	4 / 84	0.006	0.005	0.072	2 / 56	0.004
Interferon Signaling	9 / 401	0.027	0.006	0.078	10 / 69	0.005
CD163 mediating an anti- inflammatory response	2 / 14	9.51E-04	0.006	0.078	1 / 9	6.80E-04
Senescence-Associated Secretory Phenotype (SASP)	4 / 91	0.006	0.007	0.082	5 / 22	0.002
Gama-carboxylation, transport, and amino-terminal cleavage of proteins	2 / 16	0.001	0.008	0.093	5 / 277	0.002

[1]http://reactome.org

[2]Differentially-regulated genes ΔPdh vs. WT. See Table S2.

[3]False discovery rate
