# Supplementary material for: Central carbon flux controls growth/damage balance for Streptococcus pyogenes
Source: PLoS Pathog. 2023 Jun 29;19(6):e1011481. doi: 10.1371/journal.ppat.1011481 (PMC10337930; doi:10.1371/journal.ppat.1011481)
Supplement: S4 Table — (PDF) [file ppat.1011481.s010.pdf]

Table S4. Plasmids used in this study

Plasmid	Features	Reference
Cloning plasmids		
pABG5	Shuttle vector	1
pJRS233	Low-copy shuttle vector used for allelic replacement	2
pGCP213	High-copy shuttle vector used for allelic replacement	3
pSPC18	Integrational vector	4
Allelic replacement plasmids[1]		
pEP98	pGCP213::*Δldh*, allelic replacement plasmid	This study
pJAM199	pGCP213::*Δpdh*, allelic replacement plasmid	This study
pEP101	pGCP213::*Δpfl*, allelic replacement plasmid	This study
pJAM147	pGCP213::*Δslo*, allelic replacement plasmid	This study
Complementation plasmids[1]		
pJAM86	pGCP1122::*3'guaB-Pdh*, allelic replacement plasmid	This study

[1]Plasmids constructed as described in the Materials and Methods using the primers listed in Table S5.

REFERENCES

1. Granok AB, Parsonage D, Ross RP, Caparon MG. 2000. J Bacteriol. 182:1529-40.
2. Perez-Casal J, Price JA, Maguin E, Scott JR. 1993. Mol Microbiol. 8:809-19.
3. Nielsen HV, Guiton PS, et. al., 2012. MBio. 4:e00177-12.
4. Cho KH, Caparon MG. 2005. Mol Microbiol. 57:11545-56.
