# Supplementary material for: Central carbon flux controls growth/damage balance for Streptococcus pyogenes
Source: PLoS Pathog. 2023 Jun 29;19(6):e1011481. doi: 10.1371/journal.ppat.1011481 (PMC10337930; doi:10.1371/journal.ppat.1011481)
Supplement: S5 Table — (PDF) [file ppat.1011481.s011.pdf]

Table S5. Mutagentic, complementation and RT-PCR Primers used in this Study

Name	Sequence	Template	Plasmid[2]
KO pdhA 3'R	CAGAGATAGCAGCTTCTGAAATTGG	HSC5[1]	pJAM199
KO pdhA 3'F	GTAGCCTTCGAAGACGTTTGGGTT		
KO pdhA 5'R	CAAATGTTGTTCTTTAGAAACTGTTACCAT		
KO pdhA 5'f	GCTTTGCTACTTGTTTGAAGGTGT		
KOpdhA Fwd Screen	GTCAAGGTCGGTAAAACGTGAA		
KOpdhA Rev Screen	TCCCGAAGTCTCCTCCGTA		
pdhA guaB site F	GGCACAATATGTCAGCCCTTAAAGTTACTATCGAAGACGTTTGGGTTGACTAA	HSC5	pJAM86
pdhA guaB site R	GCACCAAACTATTCTGTGCACTGATAATTTAATTACGACGCTCTTTTTGATTAGCT		
pfl 5’ F (BamHI)	GGCGGATCCTTCGTGACGTGTCATTAATCAGTGGAA	HSC5	pEP101
pfl 5' R	CAGCAGCATCAACATCTGTATTGGTTTTAACAGTTGCC		
pfl 3’ F	TACAGATGTTGATGCTGCTGAAGCTATTTCAAGTAA		
pfl 3’ R (EcoRI)	GGCGAATTCGTCAGTTGCTTTACAATGCACTAAAGC		
KOpfl Fwd Screen	GACGTGTCATTAATCAGTGGAA		
KOpfl Rev Screen	GGCAGTTTTAGTGATCTTATTGC		
ldh 5' F (BamHI)	GGCGGATCCGCCACAATGACACTCATGGCATAG	HSC5	pEP98
ldh 5' R	CAAATTCTTATACTTTTTTGTGTTGTTTAGTTGCAGTCAT		
ldh 3'F	CAAAAAAGTAGAAGAATTTGCTTCTGCTGCTAAAAAC		
ldh 3' R (PstI)	GGCCTGCAGCCAAGAAGGAGTGACCATTGAAAAG		
KOldh Fwd Screen	GGCATAGTCAATGAAACTCGTT		
KOldh Rev Screen	TTCAACAAACCTTACAACTACATC		
Up SLO KO FBamHI	GATATGGATCCATGTATAAGGTGCCAAAGGG	HSC5	pJAM147
Up SLO KO ROH	GCTAGCACGCGTACCTTTTTATCATTCTAAAATGTTTT		

Dn SLO KO FOH	ACGCGTGCTAGCGACTGGTTCAAGAGGTTCG
Dn SLO KO R	GATATCTCGAGAAAAGCTGTAATACCTCAAATAT
Fwd SLO KO	CATAAAAGGCTATACCCAATTG
Rev SLO KO	AGGCAGTAGCTACTCAAGC

[1]Chromosomal DNA template prepared from HSC5 (1).
[2]Primers were used to construct or confirm the plasmids listed in Table S4 as described in the Materials and Methods.

REFERENCES

1. Port GC, Paluscio E, Caparon MG. 2013. Genome Announc. e00612-00613.
